# Safety and immunogenicity of one versus two doses of the COVID-19 vaccine BNT162b2 for patients with cancer: interim analysis of a prospective observational study

**DOI:** 10.1016/S1470-2045(21)00213-8

**Published:** 2021-06

**Authors:** Leticia Monin, Adam G Laing, Miguel Muñoz-Ruiz, Duncan R McKenzie, Irene del Molino del Barrio, Thanussuyah Alaguthurai, Clara Domingo-Vila, Thomas S Hayday, Carl Graham, Jeffrey Seow, Sultan Abdul-Jawad, Shraddha Kamdar, Elizabeth Harvey-Jones, Rosalind Graham, Jack Cooper, Muhammad Khan, Jennifer Vidler, Helen Kakkassery, Shubhankar Sinha, Richard Davis, Liane Dupont, Isaac Francos Quijorna, Charlotte O'Brien-Gore, Puay Ling Lee, Josephine Eum, Maria Conde Poole, Magdalene Joseph, Daniel Davies, Yin Wu, Angela Swampillai, Bernard V North, Ana Montes, Mark Harries, Anne Rigg, James Spicer, Michael H Malim, Paul Fields, Piers Patten, Francesca Di Rosa, Sophie Papa, Timothy Tree, Katie J Doores, Adrian C Hayday, Sheeba Irshad

**Affiliations:** a The Francis Crick Institute, London, UK; b Peter Gorer Department of Immunobiology, School of Immunology and Microbial Sciences, King's College London, London, UK; c UCL Cancer Institute, University College London, London, UK; d Comprehensive Cancer Centre, School of Cancer & Pharmaceutical Sciences, King's College London, London, UK; e Breast Cancer Now Research Unit, King's College London, London, UK; f Department of Infectious Diseases, School of Immunology and Microbial Sciences, King's College London, London, UK; g Guy's and St Thomas' NHS Foundation Trust, London, UK; h Department of Haematological Medicine, King's College Hospital, London, UK; i Department of Plastic and Reconstructive Surgery, Royal Free NHS Foundation Trust, London, UK; j Regeneration Group, Wolfson Centre for Age-Related Diseases, Institute of Psychiatry, Psychology & Neuroscience, King's College London, London, UK; k Clinical Trials Unit, King's College London, London, UK; l Institute of Molecular Biology and Pathology, National Research Council of Italy, Rome, Italy

## Abstract

**Background:**

The efficacy and safety profiles of vaccines against SARS-CoV-2 in patients with cancer is unknown. We aimed to assess the safety and immunogenicity of the BNT162b2 (Pfizer–BioNTech) vaccine in patients with cancer.

**Methods:**

For this prospective observational study, we recruited patients with cancer and healthy controls (mostly health-care workers) from three London hospitals between Dec 8, 2020, and Feb 18, 2021. Participants who were vaccinated between Dec 8 and Dec 29, 2020, received two 30 μg doses of BNT162b2 administered intramuscularly 21 days apart; patients vaccinated after this date received only one 30 μg dose with a planned follow-up boost at 12 weeks. Blood samples were taken before vaccination and at 3 weeks and 5 weeks after the first vaccination. Where possible, serial nasopharyngeal real-time RT-PCR (rRT-PCR) swab tests were done every 10 days or in cases of symptomatic COVID-19. The coprimary endpoints were seroconversion to SARS-CoV-2 spike (S) protein in patients with cancer following the first vaccination with the BNT162b2 vaccine and the effect of vaccine boosting after 21 days on seroconversion. All participants with available data were included in the safety and immunogenicity analyses. Ongoing follow-up is underway for further blood sampling after the delayed (12-week) vaccine boost. This study is registered with the NHS Health Research Authority and Health and Care Research Wales (REC ID 20/HRA/2031).

**Findings:**

151 patients with cancer (95 patients with solid cancer and 56 patients with haematological cancer) and 54 healthy controls were enrolled. For this interim data analysis of the safety and immunogenicity of vaccinated patients with cancer, samples and data obtained up to March 19, 2021, were analysed. After exclusion of 17 patients who had been exposed to SARS-CoV-2 (detected by either antibody seroconversion or a positive rRT-PCR COVID-19 swab test) from the immunogenicity analysis, the proportion of positive anti-S IgG titres at approximately 21 days following a single vaccine inoculum across the three cohorts were 32 (94%; 95% CI 81–98) of 34 healthy controls; 21 (38%; 26–51) of 56 patients with solid cancer, and eight (18%; 10–32) of 44 patients with haematological cancer. 16 healthy controls, 25 patients with solid cancer, and six patients with haematological cancer received a second dose on day 21. Of the patients with available blood samples 2 weeks following a 21-day vaccine boost, and excluding 17 participants with evidence of previous natural SARS-CoV-2 exposure, 18 (95%; 95% CI 75–99) of 19 patients with solid cancer, 12 (100%; 76–100) of 12 healthy controls, and three (60%; 23–88) of five patients with haematological cancers were seropositive, compared with ten (30%; 17–47) of 33, 18 (86%; 65–95) of 21, and four (11%; 4–25) of 36, respectively, who did not receive a boost. The vaccine was well tolerated; no toxicities were reported in 75 (54%) of 140 patients with cancer following the first dose of BNT162b2, and in 22 (71%) of 31 patients with cancer following the second dose. Similarly, no toxicities were reported in 15 (38%) of 40 healthy controls after the first dose and in five (31%) of 16 after the second dose. Injection-site pain within 7 days following the first dose was the most commonly reported local reaction (23 [35%] of 65 patients with cancer; 12 [48%] of 25 healthy controls). No vaccine-related deaths were reported.

**Interpretation:**

In patients with cancer, one dose of the BNT162b2 vaccine yields poor efficacy. Immunogenicity increased significantly in patients with solid cancer within 2 weeks of a vaccine boost at day 21 after the first dose. These data support prioritisation of patients with cancer for an early (day 21) second dose of the BNT162b2 vaccine.

**Funding:**

King's College London, Cancer Research UK, Wellcome Trust, Rosetrees Trust, and Francis Crick Institute.

**Research in context**

**Evidence before this study**

Following SARS-CoV-2 infection, some patients with cancer, especially those with haematological malignancies, had sustained immune dysregulation, inefficient seroconversion, and prolonged viral shedding. To identify studies reporting on immunological responses to COVID-19 vaccines in patients with cancer, we searched PubMed for articles published in English between Jan 1 and Dec 1, 2020, using the search terms (“cancer” or “malignancy”) AND (“Vaccine” OR “mRNA vaccine”) AND (“COVID-19” OR “coronavirus” OR “SARS-CoV-2”). However, exclusion of patients with cancer and, in particular, those receiving systemic anticancer therapies from the registry trials of the five approved COVID-19 vaccines raised questions about the efficacy and safety of vaccination against SARS-CoV-2 in this patient population. Additionally, although the change in the UK's dosing interval to 12 weeks between vaccine doses was implemented to maximise vaccine coverage in the general population, it is unclear whether this strategy is appropriate for patients with cancer and those on systemic anticancer therapies.

**Added value of this study**

In this prospective observational study of 151 patients with solid and haematological cancers, we provide the first insights, to our knowledge, into the antibody and T-cell responses to the mRNA-based SARS-CoV-2 BNT162b2 vaccine, as well as its safety, in an immunocompromised patient population. We also assess the consequence of using different dosing schedules in this population. In patients with cancer, one 30 μg dose of the BNT162b2 vaccine yields poor efficacy, as measured by seroconversion rates, viral neutralisation capacity, and T-cell responses, at 3 weeks and 5 weeks following the first inoculum. Immunogenicity increased significantly in patients with solid cancer within 2 weeks of a vaccine boost at day 21 after the first dose.

**Implications of all the available evidence**

Our data support prioritisation of patients with cancer for an early (day 21) second dose of the BNT162b2 vaccine. Given the globally poor responses to vaccination in patients with haematological cancers, post-vaccination serological testing and careful follow-up should be prioritised for these patients, together with vaccination of those in close contact with them, in order to promote herd immunity.

## Introduction

As part of the UK's programme to control the spread of SARS-CoV-2, the Medicines and Healthcare products Regulatory Agency (MHRA) authorised the SARS-CoV-2 mRNA vaccine BNT162b2 (produced by Pfizer–BioNTech; Mainz, Germany) on Dec 2, 2020, for active immunisation to prevent COVID-19 in individuals aged 16 years and older.^1^ Since immunocompetence can be jeopardised by malignancy or its treatment, or both,^2^, ^3^, ^4^ patients with cancer were among the groups prioritised for vaccination.

The phase 3 trial of the BNT162b2 vaccine showed 95% efficacy in preventing COVID-19, including severe disease.^5^ However, of the 18 860 vaccinated individuals in the trial, none with an active oncological diagnosis was included. Exclusion criteria included a medical history of COVID-19, treatment with immunosuppressive therapy, or diagnosis of an immunocompromising condition. On Dec 30, 2020, the UK Government announced that for all priority groups, second doses of the COVID-19 vaccines should be given after approximately 12 weeks rather than 3–4 weeks as was initially recommended by the manufacturing license. On Feb 12, 2021, the UK Government's Green Book, chapter 14a, was updated with a note of caution about possible low vaccine responses in immunosuppressed patients, and a recommendation that, where possible, the second dose of the COVID-19 vaccine should be scheduled earlier.^6^

We recently reported that, following SARS-CoV-2 infection, some patients with cancer, particularly those with B-cell malignancies, showed delayed or negligible seroconversion, prolonged virus shedding, and sustained immune-dysregulation, compared to individuals without cancer.^7^, ^8^ Likewise, suboptimal vaccine efficacy has been reported in older and immunocompromised populations.^9^, ^10^, ^11^, ^12^ The prospect that patients with cancer might be wholly or partially unprotected by vaccination has implications for their health and for the control of SARS-CoV-2 transmission within their environments, including health-care facilities.^7^, ^13^, ^14^ Consequently, we launched the SOAP-02 vaccine study to address the safety and efficacy of the BNT162b2 vaccine in patients with cancer, and to investigate whether or not a boost at day 21 following initial vaccination was beneficial in this patient population.

## Methods

### Study design and participants

We did a prospective, longitudinal observational study of patients with cancer. Between Dec 8, 2020, and Feb 18, 2021, patients with a known diagnosis of cancer presenting at three hospitals (Guy's & St Thomas' NHS Trust, King's College Hospital, and Princess Royal University Hospital) in London, UK, who were eligible for the BNT162b2 vaccine, were screened and approached for inclusion in the SOAP-02 study (appendix p 25). Participants gave written informed consent. We also included a cohort of prioritised healthy controls from the same three hospitals (mostly health-care workers) who were included not as a control cohort for patients with cancer (who were mostly older in age), but to facilitate comparisons of vaccine immunogenicity and safety in our study with other studies of healthy cohorts receiving BNT162b2.^5^, ^15^, ^16^ The study was approved by the institutional review boards of the participating institutions (IRAS ID 282337, REC ID 20/HRA/2031).

### Procedures

Blood samples were captured before vaccination (timepoint 1), at week 3 after the first vaccine dose (timepoint 2), and at week 5 after the first vaccine dose (timepoint 3; appendix p 9). Follow-up is planned for further blood sampling after the delayed (12-week) vaccine boost. Where possible, serial nasopharyngeal SARS-CoV-2 real-time RT-PCR (rRT-PCR) swab tests were taken every 10 days or in cases of symptomatic COVID-19. This is because in order to assess the immune efficacy of BNT162b2, it was essential to exclude individuals whose immune systems might have been stimulated by past or concurrent infection. Telephone consultations to evaluate reactogenicity and safety were scheduled weekly where possible. Adverse events were graded according to the following scale: grade 1 (mild; does not interfere with activity); grade 2 (moderate; interferes with activity), grade 3 (severe; prevents daily activity), and grade 4 (potentially life-threatening; emergency department visit or admission to hospital). The interim results of safety and immunogenicity for participants up to timepoint 3 are reported here using data obtained up to March 19, 2021. Participants vaccinated between Dec 8 and Dec 29, 2020, received two 30 μg doses of BNT162b2 administered intramuscularly 21 days apart, but in line with changes to national government guidelines on Dec 30, 2020, patients vaccinated after this date received only one dose within the study period, with a planned follow-up boost at 12 weeks. Full details of the inclusion and exclusion criteria are in the protocol (appendix p 26).

Details of laboratory analyses done on blood samples are described in the appendix (pp 2–3, 8, 18–24). Briefly, the immunogenicity of BNT162b2 was assessed by ELISA for antibody seroconversion; by neutralisation assays of the ancestral SARS-CoV-2 Wuhan strain and of a variant of concern (spike variant of concern 202012/01, lineage B.1.1.7; Kent, UK); by fluorospot assays for interferon-γ (IFNγ)-producing and interleukin-2 (IL-2)-producing SARS-CoV-2-reactive T cells; and by flow cytometry phenotyping of peripheral blood mononuclear cells. As described previously,^17^ we deployed ELISA to measure IgG antibodies specific for the SARS-CoV-2 spike (S) protein included in the vaccine. The test was validated for batch effects and standardised with positive and negative controls, setting a positive threshold of 70 EC_50_ dilution units. Baseline scores greater than 70 provided an additional means to identify individuals with past or concurrent SARS-CoV-2 exposure, added to which we used an ELISA for baseline or developing IgG reactivity, or both, to the SARS-CoV-2 nucleocapsid (N) protein not included in the vaccine. This ELISA has also been described previously,^17^, ^18^ and was re-validated. As previously described,^17^, ^18^, ^19^ plasma samples were incubated with wild-type or B1.1.7 pseudotyped virus and HeLa cells stably expressing the ACE2 receptor. Infection level was assessed in lysed cells. The fluorospot assays (appendix pp 12–13) were used to quantitate T cells secreting IFNγ or IL-2, or both, in response to stimulation with two separate SARS-CoV-2 S protein peptide pools, one spanning the S2 domain and one spanning the receptor binding domain (RBD), which is not contained within S2.^15^, ^20^ Peptides within these pools can stimulate MHC class I-restricted and MHC class II-restricted T cells.^20^, ^21^ Additionally, we compared responses to peptides derived from commonly encountered viruses (cytomegalovirus, Epstein-Barr virus, and influenza) and from tetanus. The assay was stringently assessed for batch effects by use of validated positive and negative controls (appendix pp 12–13). For all assays, a single cutoff for T-cell responses of more than seven cytokine-secreting cells per 10^6^ peripheral blood mononuclear cells was determined by receiver operated characteristic (ROC) analysis (appendix pp 12–13). Individuals scoring greater than seven for IFNγ or IL-2 or both, in response to RBD or S2 peptide pools, or both, were classified as responders.

### Outcomes

The coprimary endpoints were seroconversion to the SARS-CoV-2 S protein in patients with cancer following single-dose vaccination with BNT162b2, and the effect of vaccine boosting 21 days later on seroconversion. The secondary endpoints were safety following each vaccine dose, T-cell responses, and neutralisation of the SARS-CoV-2 Wuhan strain and of the variant of concern B.1.1.7 (Kent). Follow-up blood sampling is planned after the delayed boost for study participants who were not boosted at day 21.

### Statistical analysis

The sample size for the interim analysis was not based on statistical hypothesis testing. All participants with available data were included in the safety and immunogenicity analyses. Samples were immediately assigned an ID upon receipt, and sample processing and analysis was done without any experimental operator knowing the nature of the sample, consistent with good laboratory practice. Samples were categorised as healthy controls, solid cancers, and haematological cancers; by timepoints 1, 2, or 3; and into boost and non-boost groups. Statistics were computed in R, version 1.2.5042 (R Core Team 2020), using the rstatix package (version 0.7.0). The significance threshold for p values was less than 0·05 after correction for multiple comparisons. The proportion of responders above the threshold and 95% CIs calculated by the Wilson method are reported. The effect of boosting on serological response at timepoint 3 was assessed by Fisher's exact tests, and p values were corrected by the Benjamini-Hochberg method to correct for testing of multiple parameters derived from the same assay: neutralisation results were corrected for two comparisons (wild-type and B.1.1.7 strains), flow cytometry cell counts were corrected for two comparisons (T cells and B cells), and fluorospot results were corrected for six comparisons (IFNγ^+^, IL-2^+^, and IFNγ^+^IL-2^+^ T-cell responses to each of the receptor-binding domain [RBD] and S2 peptide pools). DMSO (dimethyl sulfide; negative) and CEF/CEFT (cytomegalovirus, Epstein-Barr virus, influenza virus [and tetanus toxin] peptide pools; positive) fluorospot control results were not considered in the correction. Additionally, the Benjamini-Hochberg correction was applied to paired Wilcoxon tests run in parallel on different cancer types and boost versus no-boost cohorts when assessing patient trajectories between timepoints 2 and 3.

The study is registered with the NHS Health Research Authority and Health and Care Research Wales (REC ID 20/HRA/2031).

### Role of the funding source

The academic authors retained editorial control. None of the funders of the study had any role in study design, data collection, data analysis, data interpretation, or writing of the report.

## Results

151 patients with cancer (95 patients with solid cancer and 56 patients with haematological cancer) and 54 healthy controls were enrolled into the SOAP-02-vaccine study. The clinical characteristics of study participants are summarised in table 1.

**Table 1:** Clinical characteristics of patients with cancer and healthy controls

			Patients with cancer (n=151)*	**Healthy controls (n=54)**
Median age, years (IQR)			73·0 (64·5–79·5)	40·5 (31·3–50·0)
Sex				
	Male		78/151 (52%)	28/54 (52%)
	Female		73/151 (48%)	26/54 (48%)
Race				
	White		124/151 (82%)	33/54 (61%)
	Black, Asian, and minority ethnic		27/151 (18%)	21/54 (39%)
Non-oncological comorbidities				
	Cardiovascular disease (ischaemic heart disease, hypertension, hypercholesteraemia)		62/151 (41%)	0
	Diabetes		22/151 (15%)	0
	Underlying lung pathology		12/151 (8%)	0
	None of the above		55/151 (36%)	0
Solid malignancies				
	Women's cancers (gynaecological, breast)		33/95 (35%)	NA
	Urological cancers (renal, prostate, testicular, bladder)		15/95 (16%)	NA
	Skin cancers (melanoma, Merkel cell carcinoma)		12/95 (13%)	NA
	Thoracic malignancies (lung, mesothelioma)		21/95 (22%)	NA
	Gastrointestinal cancers (stomach, oesophageal, pancreas, colorectal)		12/95 (13%)	NA
	Head and neck cancer		1/95 (1%)	NA
	Glioblastoma		1/95 (1%)	NA
Haematological malignancies				
	Mature B-cell neoplasms		38/56 (68%)	NA
		Chronic lymphocytic leukaemia or small lymphocytic lymphoma	11/38 (29%)	NA
		Plasma cell myeloma	9/38 (24%)	NA
		Diffuse large B cell lymphoma	8/38 (21%)	NA
		Follicular lymphoma	4/38 (11%)	NA
		Lymphoplasmacytic lymphoma	1/38 (3%)	NA
		Burkitt's lymphoma	1/38 (3%)	NA
		Mantle cell lymphoma	1/38 (3%)	NA
		MALT lymphoma	1/38 (3%)	NA
		Nodular sclerosing Hodgkin lymphoma	1/38 (3%)	NA
		Post-renal transplant lymphoproliferative disorder	1/38 (3%)	NA
	Mature T-cell neoplasms		5/56 (9%)	NA
		Anaplastic large cell lymphoma	4/5 (80%)	NA
		Angioimmunoblastic T-cell lymphoma	1/5 (20%)	NA
	Myeloid and acute leukaemia neoplasms		10/56 (18%)	NA
		Acute myeloid leukaemia	3/10 (30%)	NA
		Myelodysplastic syndrome or myeloproliferative neoplasms	2/10 (20%)	NA
		Chronic myelomonocytic leukaemia	2/10 (20%)	NA
		T-cell precursor acute lymphoblastic leukaemia	2/10 (20%)	NA
		Myelofibrosis	1/10 (10%)	NA
	Others		3/56 (5%)	NA
		Osteomyelofibrosis	1/3 (33%)	NA
		Amyloid light-chain amyloidosis	1/3 (33%)	NA
		Erdheim-Chester disease	1/3 (33%)	NA
TNM staging (solid tumours only)				
	I		8/95 (8%)	NA
	II		6/95 (6%)	NA
	III		26/95 (27%)	NA
	IV		54/95 (57%)	NA
	Missing data		1/95 (1%)	NA
Time from cancer diagnosis to study recruitment				
	<3 months		34/151 (23%)	NA
	3 to <12 months		30/151 (20%)	NA
	12–24 months		24/151 (16%)	NA
	>24 months		53/151 (35%)	NA
	Missing data		10/151 (7%)	NA

Data are n/N (%), unless otherwise specified. NA=not applicable. MALT=mucosa-associated lymphoid tissue.

* 95 patients with solid malignancies and 56 with haematological malignancies.

All 151 patients were vaccinated with BNT162b2 on day 1. Thereafter, 25 patients with solid cancer and six patients with haematological cancer received a second dose on day 21. 69 patients with solid cancer and 49 patients with haematological cancer have been scheduled for a delayed boost at around 12 weeks, and two patients (one with solid cancer and one with haematological cancer) died during the study period; both deaths were related to COVID-19 (table 2). 16 healthy controls received two doses 21 days apart, while a 12-week boost is planned for the remaining 38. As might be expected in a longitudinal cohort study done while the variant of concern B.1.1.7 was highly prevalent in England,^22^ coupled with a national lockdown commencing Jan 4, 2021,^23^ we observed sample attrition at different study junctures, as outlined in the appendix (p 4) and table 2. Accommodating attritions, 736 blood samples were processed for assessing the immunogenicity of BNT162b2, distributed as indicated across the different outcome measures (table 2). In individuals assayed for anti-SARS-CoV-2 IgG responses, some were assessed for virus neutralisation or T-cell responses, or both (table 2). Median follow-up times from first vaccination to blood sample analysis were 22 days (IQR 19–27) for healthy controls at timepoint 2, 22 days (21–26) for patients with solid and haematological cancers at timepoint 2; and 40 days (36–42) for healthy controls at timepoint 3, 37 days (35–42) for patients with solid cancer at timepoint 3, and 37 days (35–40) for patients with haematological cancers at timepoint 3 (appendix p 4). Analyses of samples and data obtained after March 19, 2021, are ongoing.

**Table 2:** Overall study population and available number of samples for assessment of each study outcome

		**Cancer cohort (n=151)**		**Healthy controls (n=54)**
		Solid cancers (n=95)	Haematological cancers (n=56)	
**Overall study population**				
Received first dose		95/95 (100%)	56/56 (100%)	54/54 (100%)
Received day 21 boost		25/95 (26%)	6/56 (11%)	16/54 (30%)
Awaiting delayed second dose boost		69/95* (73%)	49/56*(88%)	38/54 (70%)
**Study outcome**				
Anti-SARS-CoV-2 IgG response				
	Pre-vaccination baseline samples	55/95 (58%)	34/56 (61%)	12/54 (22%)
	First-dose efficacy at week 3	56/95 (59%)	44/56 (79%)	34/54 (63%)
	Efficacy at week 5: no boost	33/95 (35%)	36/56 (64%)	21/54 (39%)
	Efficacy at week 5 after day 21 boost	19/95 (20%)	5/56 (9%)	12/54 (22%)
Neutralisation assays				
	First-dose efficacy at week 3	54/95 (57%)	39/56 (70%)	32/54 (59%)
	Efficacy at week 5: no boost	21/95 (22%)	25/56 (45%)	18/54 (33%)
	Efficacy at week 5 after day 21 boost	25/95 (26%)	5/56 (9%)	12/54 (22%)
T-cell vaccine response				
	Pre-vaccination baseline samples	4/95 (4%)	3/56 (5%)	2/54 (4%)
	First-dose efficacy at week 3	31/95 (33%)	18/56 (32%)	17/54 (31%)
	Efficacy at week 5: no boost	15/95 (16%)	18/56 (32%)	13/54 (24%)
	Efficacy at week 5 after day 21 boost	16/95 (17%)	4/56 (7%)	3/54 (6%)
Seropositive or SARS-CoV-2 swab positive				
	Excluded from overall immune efficacy analysis	9/95 (9%)	3/56 (5%)	5/54 (9%)
Adverse events				
	Following first dose	90/95 (95%)	50/56 (89%)	40/54 (74%)
	Following week 3 booster	25/25 (100%)	6/6 (100%)	16/16 (100%)

Data are n/N (%).

* Two COVID-19-related deaths before receiving the second dose of the vaccine.

The distribution of anticancer treatments given in relation to the date of vaccine administration for patients with solid and haematological cancers is shown in the appendix (pp 5–6). 38 (41%) of 92 patients with solid cancer had anticancer treatment within 15 days preceding day 1 vaccination; and 50 (54%) of 92 received anticancer treatment within 15 days after day 1 vaccination. Among those receiving a boost, nine (36%) of 25 patients with solid cancer received anticancer treatments within 15 days before the vaccine, as did 15 (60%) of 25 patients within 15 days after the vaccine (appendix pp 5–6). 26 (47%) of 55 patients with haematological cancer received anticancer treatments within 15 days preceding day 1 vaccination, and 27 (49%) of 55 received anticancer treatment within 15 days after day 1 vaccination (appendix pp 5–6). Two (33%) of six patients with haematological cancer who received a boost had anticancer treatment within 15 days of vaccination.

Owing to sample attrition, only 79 patients were able to attend for screening for asymptomatic SARS-CoV-2 infections during the study period, and only 12 provided multiple swabs. Up until day 21 after the first vaccine inoculum, six positive cases of SARS-CoV-2 infection were identified (appendix pp 10–11), and two patients died from COVID-19, one before blood sampling at timepoint 2. No new positive swab tests were recorded more than 21 days after vaccination (appendix pp 10–11).

By testing for antibody seroconversion, we identified five healthy controls, seven patients with solid cancer, and three patients with haematological cancer as possibly having previous SARS-CoV-2 exposure, and two (one healthy control and one patient with solid cancer) who were confirmed to have had SARS-CoV-2 infection by the swab test. Thus, these 15 participants, and the two patients with solid cancer who were swab-positive and seronegative, were removed from the cohort analysis of immunogenicity (table 2), but their immune reactivity towards the SARS-CoV-2 S protein is considered below.

**Figure 1:**
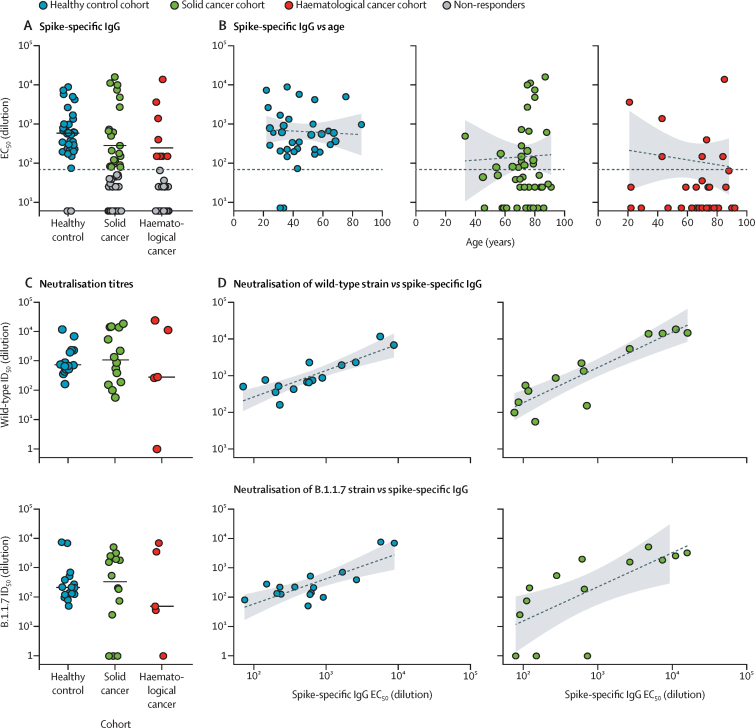
Serological response to COVID-19 vaccine BNT162b2 (A) Spike-specific IgG titres (EC_50_) in plasma samples at 3 weeks after the vaccine in serological responders: healthy controls (n=32), patients with solid cancers (n=21), and patients with haematological cancer (n=8). The horizontal line represents the threshold of specific response. Short bars represent the median values of responder values only. Sample comparisons tested by Kruskal-Wallis with Dunn's post-hoc test on responder values only; no significant differences. (B) Association of age with serological response (spike-specific IgG ELISA) at 3 weeks after the vaccine (Spearman's correlation) in healthy controls (n=34; *r*=–0·1, p=0·58), patients with solid cancers (n=56; *r*=–0·12, p=0·47), and patients with haematological cancers (n=44; *r*=–0·11, p=0·66). The horizontal line represents the threshold of specific response. Dashed lines represent regression lines. Shading represents 95% CIs. (C) Neutralisation titres against wild-type SARS-CoV-2 (upper panel) and the B.1.1.7 variant of concern (lower panel) in plasma samples at 3 weeks after the vaccine in healthy controls (n=16), patients with solid cancer (n=14), and patients with haematological cancer (n=5). Short bars represent median values of responder values only. Sample comparisons tested by Kruskal-Wallis with Dunn's post-hoc test, corrected by Benjamini-Hochberg method; no significant differences. (D) Correlation between spike-specific IgG titres and neutralisation titres against wild-type SARS-CoV-2 samples (upper panels) at 3 weeks after the vaccine (Spearman's correlation) in healthy controls (n=16; *r*=0·18, p=0·00025) and patients with solid cancer (n=14; *r*=0·84, p=0·00028). Correlation between spike-specific IgG titres and neutralisation titres against B.1.1.7 SARS-CoV-2 samples (lower panels) at 3 weeks after the vaccine (Spearman's correlation) in healthy controls (n=16; *r*=0·55, p=0·030) and in patients with solid cancer (n=14; *r*=0·84, p=0·026). Dashed lines represent regression lines. Shading represents 95% CIs. EC_50_=50% effective concentration. ID_50_=inhibitory dilution at which 50% of viral particles are neutralised.

When the remaining 134 individuals were examined for anti-S IgG titres at approximately 21 days following vaccination, 32 (94%) of 34 healthy controls, 21 (38%) of 56 patients with solid cancer, and eight (18%) of 44 patients with haematological cancer were classified as responders (table 3). Maximum anti-S IgG titres were approximately 100 times higher than minimum responses, but median titres were largely similar in each cohort (figure 1A). Thus, the main difference between healthy controls and patients with cancer was a failure to produce a response, rather than the magnitude of the response. Failure to produce a response to first-dose vaccination was not obviously attributable to age (figure 1B); indeed, when responses were parsed into those above threshold (>70 units), those below threshold (25–70 units), and those below the limit of detection (<25 units), the age distribution for 21 patients with solid cancer registering as above threshold was similar to that of 17 patients classified as below the limit of detection (appendix pp 10–11).

**Table 3:** Immunogenicity of BNT162b2 vaccine

	**First-dose immunogenicity at week 3 (95% CI)**	**Immunogenicity at week 5 (95% CI)**	
		No boost	Day 21 boost
**Anti-SARS-CoV-2 IgG response**			
Health-care workers	32/34; 94% (81–98)	18/21; 86% (65–95)	12/12; 100% (76–100)
Solid cancer cohort	21/56; 38% (26–51)	10/33; 30% (17–47)	18/19; 95% (75–99)
Haematological cancer cohort	8/44; 18% (10–32)	4/36; 11% (4–25)	3/5*; 60% (23–88)
**T-cell vaccine response**			
Health-care workers	14/17; 82% (59–94)	9/13; 69% (42–87)	3/3*; 100% (44–100)
Solid cancer cohort	22/31; 71% (53–84)	8/15; 53% (30–75)	14/16; 88% (64–97)
Haematological cancer cohort	9/18; 50% (29–71)	6/18; 33% (16–56)	3/4*; 75% (40–95)

Data are n/N; % (95% CI). 95% CIs were calculated by the Wilson method.

* Insufficient numbers for clinical interpretation.

The functional implications of seroconversion were assessed by neutralisation of infection by either the SARS-CoV-2 Wuhan strain (referred to as wild type, or as England 2020/02/407073), which was pre-eminent in the UK during most of 2020 and which is matched to BNT162b2, or by the spike variant of concern B.1.1.7 (Kent), which was highly prevalent during the SOAP-02 study period.^22^ We used a recently described neutralisation assay^17^, ^18^, ^19^ and found that all responders, with the exception of a single responder with haematological cancer, could neutralise the wild-type strain (figure 1C). Conversely, an additional three responders with solid cancer could not neutralise the variant of concern B.1.1.7 strain; moreover, in healthy controls, neutralisation titres for the variant of concern B.1.1.7 strain were significantly lower (p=0·0010) than for the wild-type strain (figure 1C; appendix pp 10–11). Anti-S IgG titres and neutralisation correlated strikingly for healthy controls and for patients with solid cancer (figure 1D), but too few patients with haematological cancer seroconverted to facilitate this comparison in this cohort.

In sum, a single dose of 30 μg BNT162b2 failed to induce seroconversion in most patients with cancer. Although assumptions are commonly made about immunodeficiencies in patients with cancer, particularly those with haematological cancers, in whom immunogenicity was particularly poor, we noted no significant correlation of blood B-cell or T-cell counts with responder or non-responder status (appendix pp 10–11).

**Figure 2:**
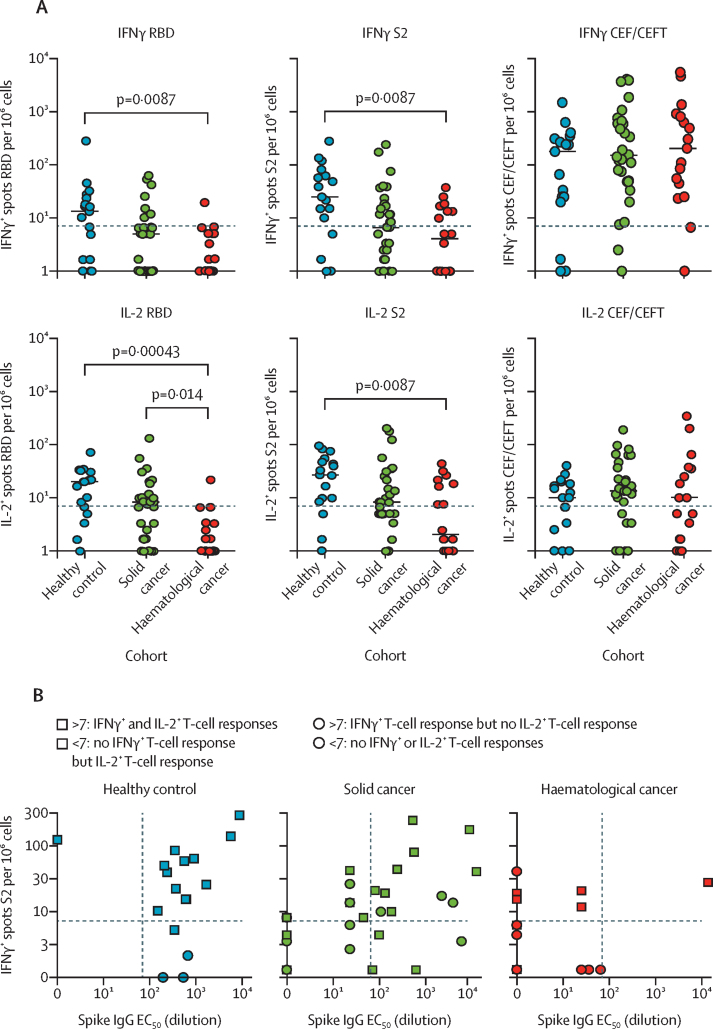

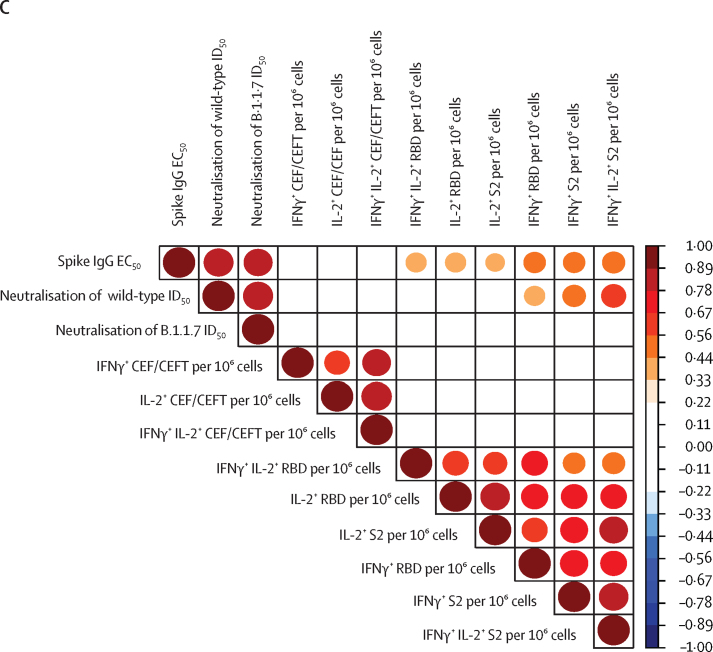
T-cell response to COVID-19 vaccine BNT162b2 (A) IFNγ^+^ and IL-2^+^ responses to stimulation with peptides from RBD, S2, and CEF/CEFT reported as number of spots per 10^6^ cells in PBMC samples at 3 weeks after the vaccine in healthy controls (n=17), patients with solid cancer (n=31), and patients with haematological cancer (n=18). Short bars represent median values for each group; the horizontal line represents the threshold of specific response. Kruskal-Wallis test with Dunn's post-hoc test, corrected by Benjamini-Hochberg method. p values shown where inter-group comparisons were significant: healthy control versus haematological cancer cohorts (IFNγ RBD, IFNγ S2, IL-2 RBD, IL-2 S2) and solid cancer versus haematological cancer cohorts (IL-2 RBD). (B) Relationship between serological response and T-cell response in healthy controls (n=17), patients with solid cancer (n=31), and patients with haematological cancer (n=18). The horizontal lines represent thresholds of S2-specific IFNγ T-cell responses and the vertical lines represent the thresholds of S-reactive serological responses. Square data points denote S2-specific IL-2 producers (ie, IL-2 threshold of >7 spots). No statistical test was done to assess the association between serological and T-cell responses because the plot serves to highlight the responder status of patients by threshold as a graphical representation. (C) Spearman's correlation between T-cell responses (fluorospot counts per 10^6^ PBMC) and serological responses as determined by ELISA and neutralisation assays across all study participants. The colour scale indicates Spearman's *r* value; all p values are less than 0·01. The circle sizes are proportional to the correlation coefficient. CEF/CEFT=cytomegalovirus, Epstein-Barr virus, influenza virus (and tetanus toxin) peptide pools. EC_50_=50% effective concentration. ID_50_=inhibitory dilution at which 50% of viral particles are neutralised. IFNγ=interferon-γ. IL-2=interleukin-2. PBMC=peripheral blood mononuclear cell. RBD=receptor binding domain. S=spike protein. S2=spike protein 2.

In our fluorospot assays to assess T-cell vaccine response, of the healthy controls assayed, 14 (82%) of 17 were responders (table 3), with only three showing neither IFNγ-producing nor IL-2-producing cells (figure 2A, three blue dots at <7 in S2-reactive IL-2 plot; appendix pp 12–13). Bivariate representation showed that seroconversion correlated strongly with T-cell responses, with only one healthy control serological non-responder showing T-cell responsiveness, and three healthy control serological responders failing to show T-cell responses (figure 2B).

Of patients with solid cancer assayed, 22 (71%) of 31 were T-cell responders—a seemingly higher immune efficacy than that observed for seroconversion (table 3, figure 2A). The samples assayed for ELISA and T-cell responses were relatively well balanced for tumour types and treatment, although nine (17%) of 53 patients examined by ELISA received chemotherapy compared with five (9%) of 53 assayed for T-cell responses (appendix pp 12–13). Only one serological responder showed no T-cell reactivities, whereas eight serological non-responders were T-cell responders (figure 2B). The dynamic range of T-cell responses was broad (0 to >300) and similar to that of healthy controls (figure 2A).

Of patients with haematological cancer assayed, nine (50%) of 18 showed IFNγ-producing or IL-2-producing T cells, or both, responding to S2 peptides, which also was a higher immune efficacy than that observed for seroconversion (table 3, figure 2A). However, the dynamic ranges of the responses were invariably lower than for healthy controls and patients with solid cancer (figure 2A). Notably, this was not because patients with haematological cancer lacked T-cell competence, because the frequencies and strengths of CEF/CEFT recall responses were quantitatively similar across all three cohorts (figure 2A). Those patients who were assayed for ELISA and T-cell responses were again well balanced for cancer subtype and treatment (appendix pp 12–13); however, eight of nine patients showing T-cell responses were serological non-responders (figure 2A, B). Whereas this finding might seem to support the hypothesis that the vaccine's T-cell immune efficacy is higher than its serological immune efficacy, we noted that patients with haematological cancer who were assayed very rarely showed RBD-responsive versus S-responsive T cells (figure 2A). Given that reactivity to S2 (the sequence of which is highly conserved, with minor variations in common cold coronaviruses) is less specific for SARS-CoV-2 than is RBD reactivity, it is possible that low-level T-cell reactivities in seronegative patients with haematological cancer reflect pre-existing T-cell reactivities to common cold coronaviruses rather than those induced by the vaccine.

In sum, a priming inoculum of 30 μg of BNT162b2 induced T-cell responses to S2 and RBD in the majority of healthy controls and patients with solid cancer, although many patients with cancer were serological non-responders. When viewed across all participants, S2-dependent or RBD-dependent IFNγ responses correlated with neutralisation of wild-type SARS-CoV-2, although less strongly than seroconversion, and they did not correlate significantly with neutralisation of the variant of concern B.1.1.7 (figure 2C).

**Figure 3:**
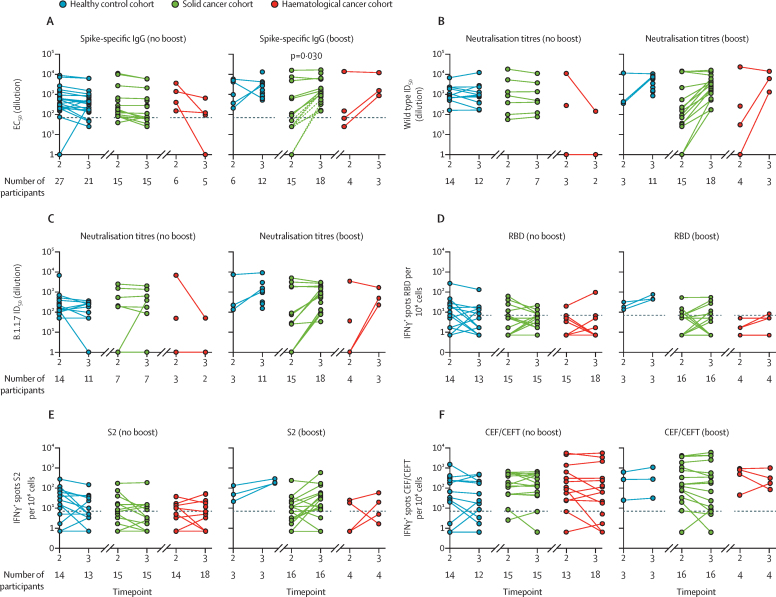
Comparison of single dose versus prime–boost with COVID-19 vaccine BNT162b2 (A) Spike-specific IgG titres in plasma samples at 3 and 5 weeks after the vaccine in individuals receiving a single vaccine dose (no boost) and in those receiving two doses (boost). Patients failing to achieve a serological response at any timepoint were excluded. Dashed lines represent eight non-responders with solid cancer at timepoint 2 who seroconverted following boost. (B) Neutralisation titres against wild-type SARS-CoV2 in plasma samples at 3 and 5 weeks after the vaccine in individuals receiving a single vaccine dose (no boost) and in individuals receiving two doses (boost). Patients failing to achieve a serological response at any timepoint were excluded. (C) Neutralisation titres against B.1.1.7 SARS-CoV-2 in plasma samples at 3 and 5 weeks after the vaccine in individuals receiving a single vaccine dose (no boost) and in individuals receiving two doses (boost). Patients failing to achieve a serological response at any timepoint are excluded. (D–F) Cytokine response to stimulation with peptides from RBD, S2, and CEF/CEFT reported as number of spots per 10^6^ PBMC at 3 and 5 weeks after the vaccine in individuals receiving a single vaccine dose (no boost) and in individuals receiving two doses (boost). All comparisons tested by paired Wilcoxon test, corrected by Benjamini-Hochberg method. CEF/CEFT=cytomegalovirus, Epstein-Barr virus, influenza virus (and tetanus toxin) peptide pools. EC_50_=50% effective concentration. ID_50_=inhibitory dilution at which 50% of viral particles are neutralised. IFNγ=interferon-γ. PBMC=peripheral blood mononuclear cell. RBD=receptor binding domain. S=spike protein. S2=spike protein 2.

We next addressed whether the primary responses to vaccination might be positively affected by boosting with 30 μg of BNT162b2. Thus, the cohorts were divided into two subcohorts—those who were boosted at day 21 and those who were not—who were then compared at timepoint 3: 5 weeks after the first dose and 14 days after the boost for those who received it. Again, some attrition occurred in blood sampling at timepoint 3 relative to timepoint 2, especially for patients who were not boosted (table 1; appendix p 4). Nonetheless, 18 (95%) of 19 patients with solid cancer were seropositive after the boost at timepoint 3, including de novo seroconversion of eight individuals (figure 3A), whereas only ten (30%) of 33 patients who were not boosted were seropositive, which was similar to the 38% seropositivity attained at week 3 following single-dose vaccination in patients with solid cancer (table 3; figure 3A). Indeed, by Fisher's exact test, the impact of boosting versus not boosting on the immune status of patients with solid cancer at timepoint 3 versus timepoint 2 was significant (p<0·0001). Moreover, boosting induced a significant increase in IgG titres (p=0·030), whereas anti-S IgG titres at timepoint 3 for those who were not boosted were either similar to those at timepoint 2 or somewhat lower (figure 3A). In healthy controls, 12 (100%) of 12 boosted participants were seropositive (table 3). In those not boosted, one had a strikingly increased titre, but anti-S IgG titres remained similar or tended to decline in the 2 weeks between timepoint 2 and timepoint 3 for most healthy controls (figure 3A).

Only six patients with haematological cancer were eligible for a boost before the change in government policy. Of these, five were analysed, of whom three (60%) were seropositive (table 3; figure 3A). Conversely, four (11%) of 36 patients analysed without boosting were seropositive at timepoint 3 (table 3), and there were some conspicuous reductions in anti-S IgG titres between timepoint 2 and timepoint 3 (figure 3A). For patients with solid cancers, the profound impact of boosting on anti-S IgG titres was mirrored by the increased capacity to neutralise both the wild-type and B.1.1.7 strains, which was similar to the results for healthy controls (figure 3B, C). The three patients with haematological cancer who were seroconverted after boosting were able to neutralise both strains (figure 3B, C), although too few were boosted for more detailed conclusions to be drawn.

A substantial quantitative impact of boosting was also apparent from assays for IFNγ-secreting T cells. Of three healthy controls, 16 patients with solid cancer, and four patients with haematological cancer who were boosted and examined for T-cell reactivities, only two patients with solid cancer and one patient with haematological cancer did not display SARS-CoV-2-peptide reactive IFNγ-producing T cells (figure 3D–E). Moreover, boosting induced the acquisition of T-cell responses in one patient with haematological cancer and four patients with solid cancer. By contrast, the general trend among healthy controls and patients with solid cancer who were not boosted was for T-cell responses to remain unaltered or commonly to decline between timepoint 2 and timepoint 3 (eg, IFNγ responses to RBD peptides; figure 3D): conversely, only two healthy controls who were seronegative at timepoint 2 showed T-cell reactivity at timepoint 3 (table 3). As a control, CEF and CEFT cell responses remained mostly the same from timepoint 2 to timepoint 3, irrespective of boosting (figure 3F). In the few patients with haematological cancer who received a boost, increased numbers of IFNγ-secreting cells were observed after boosting (figure 3D–E). There was no case of a patient who had no T-cell responses at timepoint 2 acquiring them by timepoint 3, regardless of whether or not they were boosted.

Given that most patients with cancer did not seroconvert following a primary vaccine inoculum, we investigated factors that might be associated with poor responsiveness, analysing types of malignancy, treatment, and baseline immunophenotypes that might be associated with poor responsiveness. Although the heterogeneity of the vaccine cohorts undermined statistical power in several areas (appendix pp 7–8 details specific responses at timepoint 2 and timepoint 3 by cancer subtypes), we found that serological non-responders, who comprised the most common phenotype among haematological cancers, were distributed evenly across patients with B-cell, T-cell, and myeloid-cell malignancies (appendix pp 14–15). For solid cancers as well, non-responders were spread similarly across tumour types, with some enrichment in respiratory and skin cancers (appendix pp 14–15). Non-responders were also somewhat more common among those who received the vaccine within 15 days of cancer treatment, including those receiving chemotherapy in combination with immune checkpoint inhibition (appendix pp 14–15). Only three (38%) of eight patients with solid cancer receiving checkpoint inhibitors alone were seropositive at 21 days following the first dose of the vaccine (appendix pp 14–15).

Many patients with cancer, particularly those receiving chemotherapy, regularly receive high-dose systemic steroids that can attenuate cellular immune responses and antibody production.^24^ Although there was no significant difference in the non-responder rates for patients with haematological cancer on steroid treatments compared with those not on steroids (appendix pp 14–15), serological non-responder rates were significantly enriched in patients with solid cancer who were on steroid treatment (appendix pp 14–15). Although the analysis was under-powered for significance, we also noted that of 15 patients receiving chemotherapy within 15 days of vaccination, five of whom had also received checkpoint inhibitors, the ten patients who also had concurrent high-dose dexamethasone were serological non-responders at timepoint 2 (appendix pp 14–15).

As described above, 17 individuals were excluded from the main comparisons because of suspected SARS-CoV-2 exposure. This small number of individuals did not show uniformly stronger serological or T-cell responses, with substantial heterogeneity across the dynamic ranges described for the main cohorts (appendix pp 16–17).

**Figure 4:**
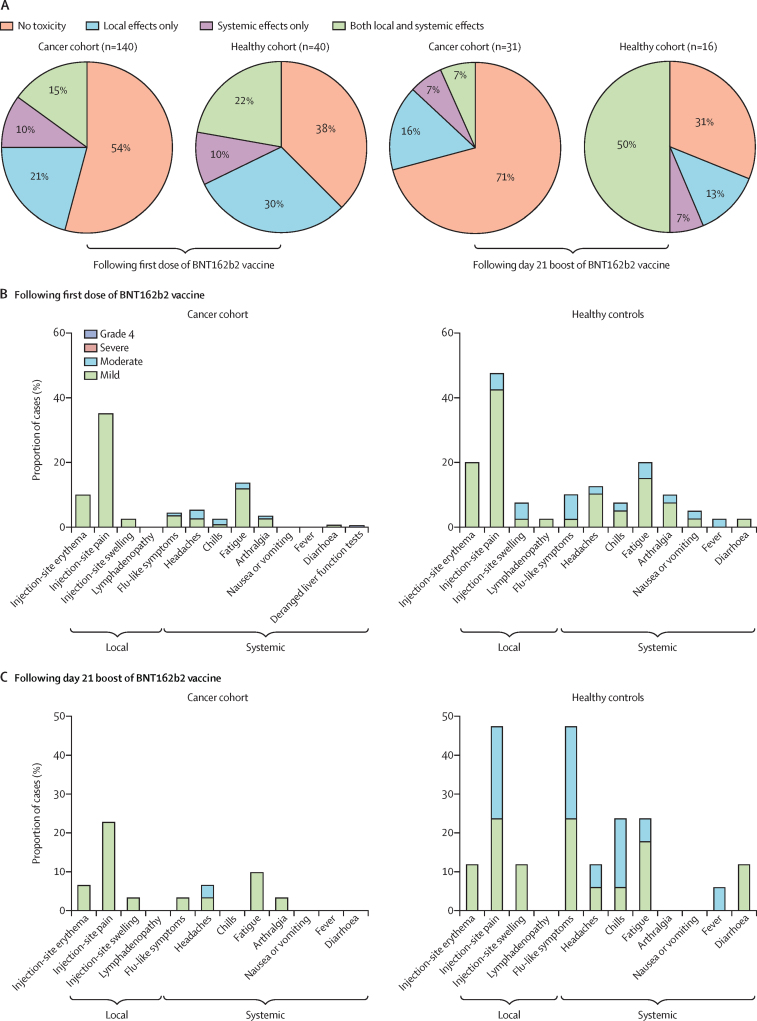
Local and systemic effects reported within 30 days after injection of COVID-19 vaccine BNT162b2 in patients with cancer and healthy controls Data on local and systemic reactions were collected via telephone consultations with participants for 30 days after vaccination. (A) Proportion of participants reporting no toxicity or toxicity (local effects only *vs* systemic effect only *vs* both local and systemic effects) following the first dose and the second booster dose of BNT162b2 on day 21. (B) Breakdown of specific local and systemic side-effects in patients with cancer and healthy controls following the first dose. (C) Breakdown of specific local and systemic side-effects in patients with cancer and healthy controls following the second booster dose of BNT162b2 on day 21. Symptoms were assessed according to the following scale: grade 1 (mild; does not interfere with activity), grade 2 (moderate; interferes with activity), grade 3 (severe; prevents daily activity), and grade 4 (potentially life-threatening; emergency department visit or admission to hospital).

Toxicity data were available for 180 participants (90 patients with solid cancer, 50 patients with haematological cancer, and 40 healthy controls) following the first dose and for 47 participants (25 patients with solid cancer, six patients with haematological cancer, and 16 healthy controls) following the boost on day 21 (table 2). 75 (54%) of 140 patients with cancer and 15 (38%) of 40 healthy controls reported no toxicities following the first dose of BNT162b2 (figure 4A). However, after boosting, 22 (71%) of 31 patients with cancer reported no toxicity compared with five (31%) of 16 healthy controls (figure 4A). Additionally, only two (7%) of 31 patients with cancer reported local and systemic effects after boosting compared with eight (50%) of 16 healthy controls (figure 4A). Injection-site pain within 7 days was the most commonly reported local reaction after the first dose of the vaccine, in 23 (35%) of 65 patients with cancer (figure 4B). Following the first and second doses, notably fewer patients with cancer reported moderate symptoms compared with healthy controls (figure 4B, C). One patient with cancer previously prescribed checkpoint inhibitors presented with deranged liver function tests requiring hospital admission (grade 4) 3 weeks following the first dose; the cause remains unclear. No notable findings emerged from routine clinical laboratory assays and patient observations (appendix p 6), and there were no differences in safety profiles between patients with haematological cancer and those with solid cancer. Safety monitoring will continue for 18 months following the vaccine boost.

## Discussion

To the best of our knowledge, this is the first report of the safety and immunogenicity of any COVID-19 vaccine in immunocompromised patient populations, specifically those with an active cancer diagnosis. The SARS-CoV-2 mRNA BNT162b2 vaccine was generally well tolerated in patients with cancer, even in those on immunotherapy who might have been anticipated to make exaggerated, inflammatory immune responses. However, by 3 weeks following single-dose (30 μg) vaccination, immunogenicity was low, 38% in patients with solid cancer and 18% in those with haematological cancer, and did not improve in the following 2 weeks. Crucially, however, each immunological metric measured was substantially improved in patients with solid cancer within 2 weeks of their receiving a boost on day 21. This improvement included seroconversion of patients with advanced-stage cancers or receiving treatments, or both, that can hinder immune responsiveness. However, the numbers of boosted patients with haematological cancer in this interim analysis were insufficient to assess the impact of boosting.

Our results are consistent with the low vaccine efficacy reported for patients with cancer receiving seasonal vaccines,^10^, ^11^, ^12^ and imply that single-dose BNT162b2 vaccination leaves most patients with cancer wholly or partially immunologically unprotected. This finding is of particular concern given our and others' observations that immunocompromised patients have a higher incidence of harbouring persistent SARS-CoV-2 infections,^7^, ^13^, ^14^ possibly providing an important reservoir for the emergence of novel viral variants.^25^, ^26^ From this perspective, a case could be made to reassess current UK policy of a 12-week BNT162b2 dosing interval in patients with cancer and other high-risk groups, in line with the update by the UK Government on Feb 12, 2021, recognising that specific populations might mount an inferior response.^6^ Additional studies examining immunogenicity after more repeated boosting of immunocompromised patients are also warranted.

Although correlates of protection against COVID-19 remain incompletely defined, vaccine immunogenicity is broadly assumed to require neutralising antibodies and antigen-specific T cells.^27^, ^28^ Our results showed that single-dose BNT162b2 induced SARS-CoV-2 S-reactive cytokine-producing T cells, neutralising IgG, or both, in more than 90% of healthy controls, which seems to be consistent with other efficacy data for this vaccine.^16^ Nonetheless, this finding does not necessarily mean that boosting has a negligible impact in healthy controls, since variables such as durable immunological memory were not measured. Moreover, it is striking that for several study participants, boosting improved neutralisation of the variant of concern B.1.1.7 strain, which is possibly germane to our concerns about the potential for variants of concern to emerge under the umbrella of incomplete immune protection.

Patients with haematological malignancies are reported to be at increased risk of adverse outcomes from SARS-CoV-2 infection,^29^ and given this vulnerability there is an urgent need to protect this population as quickly as possible. Thus, the extremely poor immune responsiveness to single-dose vaccination in this population is of particular concern. Although this interim analysis was insufficiently powered to assess the impact of the day 21 boost in these patients, it seems clear that increased measures are urgently required to induce immunological protection, most likely comprising prompt vaccine boosting and routine seroconversion monitoring. Until such measures are introduced, this population in particular should be encouraged to observe COVID-19-associated measures such as physical distancing and shielding, even after vaccination. Moreover, although patients with cancer in the UK were assigned vaccination priority level 4, no prioritisation was afforded to non-professional carers and immediate social contacts, who could potentially transmit the virus to incompletely protected patients or be infected by them. In our view, these groups should be prioritised in future pandemic planning, partly to limit the risk of increased transmission and emergence of variants of concern.

Irrespective of immunogenicity data, it could be argued that evidence for high vaccine efficacy is provided by the fact that there were no new positive swab tests from 21 days after vaccination. Moreover, it has been thought that SARS-CoV-2 infection might actually provide a boost to incompletely protected patients.^30^ We consider these viewpoints as unsubstantiated, noting that the 17 individuals in our study who were suspected of previous SARS-CoV-2 exposure did not all produce strong responses to the vaccine, and that several patients with haematological cancers who had S2-specific T-cell reactivity did not acquire T-cell reactivity to RBD. Thus, we conclude that, at the time of the change to UK policy, no information was available about the risk of changing from a planned day 21 boost to a delayed 12 week boost in patients with cancer. The decision to maximise first-dose vaccine coverage in the general population instead of prioritising clinically extremely vulnerable groups and those around them might have incurred a cost of increased risk of SARS-CoV-2 infection in these groups.

The study's limitations include insufficient power to distinguish vaccine immunogenicity in specific patient subgroups (eg, those receiving distinct treatment modalities) that differentially affect host immune responsiveness. Likewise, there was no concurrent age-matched, sex-matched, ethnicity-matched, and comorbidity-matched control group without cancer, nor a concurrent control cohort of patients with cancer who have not been vaccinated. Furthermore, given the societal setting of the vaccination campaign, convenience sampling inevitably led to missing data points for study endpoints. Large-scale collaborative consortia for assessment of vaccine responses in subpopulations are required to overcome the inherent biases of low-powered studies. Additionally, we acknowledge that multiple statistical comparisons have the potential to amplify false-positive results, although these are mostly exploratory analyses, and we invariably included p value adjustments wherever appropriate. Those limitations notwithstanding, the poor immune efficacy of single-dose BNT162b2 vaccination in patients with cancer observed in this study is abundantly clear, as is the profound positive effect of day 21 boosting in patients collectively reflecting a wide range of solid cancers and treatments. The impact of boosting on patients with haematological cancer will be determined through ongoing follow-up.

## Data sharing

Data from this study can be made available to other researchers in the field upon request and approval by the study management committee and subject to appropriate data transfer agreements. Requests should be directed to SI and ACH.

## Declaration of interests

We declare no competing interests.
