# Supplementary material for: Safety and immunogenicity of one versus two doses of the COVID-19 vaccine BNT162b2 for patients with cancer: interim analysis of a prospective observational study
Source: Lancet Oncol. 2021 Jun;22(6):765–78. doi: 10.1016/S1470-2045(21)00213-8 (PMC8078907; doi:10.1016/S1470-2045(21)00213-8)
Supplement: Supplementary appendix [file mmc1.pdf]

THE LANCET
Oncology

Supplementary appendix

This appendix formed part of the original submission and has been peer reviewed. We post it as supplied by the authors.

Supplement to: Monin L, Laing AG, Muñoz-Ruiz M, et al. Safety and immunogenicity of one versus two doses of the COVID-19 vaccine BNT162b2 for patients with cancer: interim analysis of a prospective observational study. *Lancet Oncol* 2021; published online April 27. http://dx.doi.org/10.1016/S1470-2045(21)00213-8.

Appendix

Section A: Supplementary Methods

Section B: Supplementary Tables

Section C: Supplementary Figures

Section D: Flurospot standard operating procedures

Section E: Study protocol & site recruitment file

Section A: Supplementary Methods

Participant recruitment

As a prospective, observational study set up within days of the first UK roll-out of the Pfizer vaccine, the eligibility criteria for recruitment were purposefully designed to be as inclusive as possible, thereby to obtain as much timely information on the immune responses to COVID-19 vaccines in the cancer patient population. The reported study cohort therefore consisted of patients invited for vaccination according to the UK Joint Committee on Vaccination and Immunization COVID-19 priority list and, as such, the initial recruitment included mostly older cancer patients. Following eligibility of "clinically extremely vulnerable" individuals (priority level 4) for vaccination, a younger group of cancer patients was recruited for study follow up. The primary goal of the healthy control cohort (primarily frontline health care workers) recruited into the study was to provide an experimental control for study assays, but also to facilitate the comparison of our results with other studies of BTN162b2 in healthy populations.

Laboratory analysis

Sample processing and cell isolation

Fresh whole blood samples were collected in heparin tubes (BD Biosciences) and sample processing was performed under enhanced Biosafety Level 2 conditions. Aliquots of blood from heparin tubes were stained for whole-blood flow cytometry panels (see below) or centrifuged at 2,000g for 10 min and plasma stored at −80 °C. Remaining heparinized blood was diluted with 50% volume PBS, layered over Ficoll (GE Healthcare) in Leucosep tubes (Greiner Bio-One) and centrifuged at 800g for 15 min without brake at room temperature (RT). The peripheral blood mononuclear cell (PBMC) fraction was then washed three times in cold PBS and frozen at -80C in CS10 Cryostor using MrFrosty containers before being transferred to liquid nitrogen for long-term storage.

Flow cytometry staining and acquisition

50µl of whole blood was stained in 50µl of antibody staining mix (appendix P8) and incubated for 20 minutes at RT before washing in Dulbecco-PBS and fixation for 10 min with Cellfix (BD Biosciences). Red blood cell lysis was performed twice using eBioscience 1x RBC lysis buffer for 15 min at room temperature, whereupon samples were spun down and resuspended in 200 µl of staining buffer for acquisition by flow cytometry. 100 µl of sample was analysed on a four-laser BD LSR Fortessa acquired with a BD high-throughput sampler, and populations were gated in FlowJo as described previously.[1]

ELISA protocol

ELISAs were conducted as previously described.[2] All plasma samples were heat-inactivated at 56 °C for 30 min before use. High-binding ELISA plates (Corning, 3690) were coated with antigen (Nuclear (N) protein or the S glycoprotein at 3µg ml^{-1} (25 µl per well) in PBS, either overnight at 4 °C or for 2 h at 37 °C. Wells were washed with PBS-T (PBS with 0.05% Tween-20) and then blocked with 100 µl of 5% milk in PBS-T for 1 h at room temperature. The wells were emptied and serial dilutions of plasma (starting at 1:25, 6-fold dilution) were added and incubated for 2 h at room temperature. Control reagents included CR3009 (2 µg ml^{-1})(N-specific monoclonal antibody), CR3022 (0.2 µg ml^{-1})(S-specific monoclonal antibody), negative control plasma (1:25 dilution), positive control plasma (1:50), and blank wells. Wells were washed with PBS-T. Secondary antibody was added and incubated for 1h at room temperature. IgG was detected using goat-anti-human-Fc-AP (alkaline phosphatase) (1:1,000) (Jackson, catalogue no. 109-055-098) and wells were washed with PBS-T and AP substrate (Sigma) was added and plates read at 405 nm. ELISA measurements were performed in duplicate. EC50 values were calculated in GraphPad Prism. Where an EC50 was not reached at 1:25, a plasma was considered seropositive if the OD at 405nm was 4-fold above background[2] and a value of 25 was assigned.

SARS-CoV-2 (wild-type and B.1.1.7) pseudotyped virus preparation.

Pseudotyped HIV virus incorporating the SARS-Cov-2 England 2 (England 2020/02/407073, hereafter referred to as WT) or B.1.1.7 strain Spike was prepared as previously described.[3] Pseudotyped viruses produced in a 10 cm dish seeded the day prior with 5x106 HEK293T/17 cells in 10 mL of complete Dulbecco's Modified Eagle's Medium (DMEM-C, 10% foetal bovine serum (FBS) and 1% Pen/Strep (100 IU/mL penicillin and 100 mg/mL streptomycin)). Cells were transfected using 90 mg of PEI-Max (1 mg/mL, Polysciences) with: 15 µg of HIV-luciferase plasmid, 10 µg of HIV 8.91 gag/pol plasmid and 5 µg of SARS-CoV-2 Spike protein plasmid (wild-type or B.1.1.7 in pCDNA3.1). Pseudotyped virus was harvested after 72 hours, filtered through a 0.45mm filter, concentrated by ultracentrifugation and stored at -80°C until required.

Neutralization assay with SARS-CoV-2 (wild-type and B.1.1.7).

Neutralization assays were conducted as previously described.[3] Serial dilutions of plasma samples (heat inactivated at 56 °C for 30 min) were prepared with DMEM-C media and incubated with WT or B1.1.7 pseudotyped virus for 1 h at 37 °C in 96-well plates. Next, HeLa cells stably expressing the ACE2 receptor (provided by Dr James Voss, Scripps Research, La Jolla, CA) were added (12,500 cells/50µL per well) and the plates were left for 72 hours. Infection level was assessed in lysed cells with the Bright-Glo luciferase kit (Promega), using a Victor™ X3 multilabel reader (Perkin Elmer). Measurements were performed in duplicate and the duplicates used to calculate the ID_{50} using GraphPad Prism.

Interferon-gamma (IFN-γ) and Interleukin-2 (IL-2) producer cell detection by Fluorospot

Cryopreserved PBMC from vaccinated individuals were thawed and stimulated with peptide pools spanning the receptor binding domain (RBD) or the C-terminal S2 domain of the SARS-CoV-2 spike protein (S-2), CEF (CMV, EBV, influenza virus HLA class I epitopes) + CEFT (CMV, EBV, influenza virus, tetanus toxoid HLA class II epitopes) as a positive control (JPT Peptides, final concentration of 0.25µg/ml/peptide) or DMSO, as a negative control. IFN-γ/IL-2 responses were detected by direct FluoroSpot assays (MabTech, Sweden) according to the manufacturer's instructions (appendix p 18). Briefly, after resting for 1 hour, $2x10^5$ PBMC were transferred to wells of a pre-coated FluoroSpot plate and stimulated in triplicate for 24h with the stimuli described above. Afterward, cells were removed by washing, and antibody-bound cytokine identified using a cocktail of anti-IL-2 and anti-IFN-γ detection antibodies followed by two fluorescent conjugates according to manufacturer's instructions. Plates were scanned using AID *i*Spot Spectrum reader and analysed using AID EliSpot 8.0' software (AID Autoimmun Diagnostika). Data were analysed using a QC standard operating procedure and values are expressed as cytokine secreting cells/10^6 PBMC following subtraction of values from negative control wells. For every batch, an unvaccinated control was run as a negative control and a vaccinated individual with prior SARS-CoV-2 infection was included as a positive control. A single cut-off for T cell responses of >7 cytokine secreting cells/10^6 PBMC was determined by receiver operated characteristic (ROC) analysis using pooled IFN-γ and IL-2 and responses to RBD and S2 from infection-naïve HC individuals before and 21 days following priming inoculum, establishing a threshold value as a positive response (appendix p11). Using a threshold of ≥7 cytokine secreting cells/10^6 PBMC yielded an area under the ROC curve of 0.832, a sensitivity of 65.6%, and specificity of 95.6% for any given test. Similar results were obtained for the sensitivity and specificity for each individual response when using this same threshold confirming its suitability as a single cut-off for all responses
(appendix p 8).

References

1. Lo W, Whimbey E, Elting L, Couch R, Cabanillas F, Bodey G. Antibody response to a two-dose influenza vaccine regimen in adult lymphoma patients on chemotherapy. Eur J Clin Microbiol Infect Dis 1993; 12: 778–82.
2. Graham C, Seow J, Huettner I, et al. Impact of the B.1.1.7 variant on neutralizing monoclonal antibodies recognizing diverse epitopes on SARS-CoV-2 spike. bioRxiv 2021; published online Feb 3. https://doi.org/10.1101/2021.02.03.429355 (preprint)
3. Sahin U, Muik A, Derhovanessian E, et al. COVID-19 vaccine BNT162b1 elicits human antibody and TH1 T cell responses. Nature 2020; 586: 594–99.

Section B: Supplementary Tables

Attrition of longitudinal follow up samples

	Cancer Cohort		Healthy Controls
	Solid Cancers	Haematological Cancers	
Declined bloods/missed/unable to attend hospital at:			
TP2 (week-3 post 1st dose)	34/95 (36%)	6/56 (11%)	17/54 (31%)
TP3 (week-5 post 1st dose)	32/95 (34%)	11/56 (20%)	3/54 (6%)
Withdrew consent for further blood tests during study follow up	3/95 (3%)	-	-
COVID-19 related death	1/95 (1%)	1/56 (2%)	-
Cancer-related death	1/95 (1%)	1/56 (2%)	-

Median follow up from the data of first dose of vaccination to date of sampling at TP2 and TP3

	TP2<br>Median (IQR) days	TP3<br>Median (IQR) days
Healthy Controls	22 (7.75)	40 (6.5)
Solid Cancers	22 (5)	37 (6.5)
Haematological Cancers	22 (5)	37 (5)

Anti-cancer treatments prior or after vaccination administration

**Solid Cancers (n=92)** $^{+}$			
	0-15 days	***16-29 days***	***>30-days***
**Prior to 1st dose**	***41.3% (n=38)***	***13% (n=12)***	***46% (n=42)***
Treatment naïve/no treatment	-	-	*7/42 (17%)*
Chemotherapy	*11/38 (29%)*	*7/12 (58%)*	*11/42 (26%)*
Immune Checkpoint inhibition (CPI)	*5/38 (13%)*	*2/12 (17%)*	*6/42 (14%)*
Chemotherapy + CPI	*3/38 (8%)*	*1/12 (8%)*	*2/42 (5%)*
Targeted therapies	*10/38 (26%)*	*2/12 (17%)*	*1/42 (2%)*
Endocrine therapies	*8/38 (21%)*	-	*3/42 (7%)*
Radiotherapy	*1/38 (2%)*	-	*4/42 (10%)*
Surgery	-	-	*8/42 (19%)*
**Following 1st dose**	***54% (n=50)***	***20% (n=18)***	***26% (n=24)***
Treatment naïve/no treatment	-	-	*22/24 (92%)*
Chemotherapy	*13/50 (26%)*	*2/18 (11%)*	-
Immune Checkpoint inhibition (CPI)	*8/50 (16%)*	*2/18 (11%)*	*2/24 (92%)*
Chemotherapy + CPI	*4/50 (8%)*	*1/18 (6%)*	-
Targeted therapies	*10/50 (20%)*	*3/18 (17%)*	-
Endocrine therapies	*9/50 (18%)*	*1/18 (6%)*	-
Radiotherapy	*6/50 (12%)*	*8/18 (44%)*	-
Surgery	-	*1/18 (6%)*	-
**Prior to the Day 21 2nd dose (n=25)**	***36% (n=9)***	***64% (n=16)***	
Treatment naïve/no treatment	-	*9/16 (56%)*	
Chemotherapy	*3/9 (33%)*	*1/16 (6%)*	
Immune Checkpoint inhibition (CPI)	-	*3/16 (19%)*	
Chemotherapy + CPI	-	*1/16 (6%)*	
Targeted therapies	*3/9 (33%)*	*2/16 (13%)*	
Endocrine therapy	*1/9 (11%)*	-	
Radiotherapy	*2/9 (22%)*	-	
**After the Day 21 2nd dose (n=25)**	***60% (n=15)***	***40% (n=10)***	
Treatment naïve/no treatment	-	*9/10 (90%)*	
Chemotherapy	*3/15 (20%)*	*1/10 (10%)*	
Immune Checkpoint inhibition (CPI)	*3/15 (20%)*	-	
Chemotherapy + CPI	*1/15 (7%)*	-	
Targeted therapies	*5/15 (33%)*	-	
Endocrine therapy	*1/15 (7%)*	-	
Radiotherapy	*2/15 (13%)*	-	
**Haematological Cancers (n=55)** $^{+}$			
	0-15 days	***16-29 days***	***>30-days***
**Prior to 1st dose (n=55)**	***47% (n=26)***	***11% (n=6)***	***42% (n=23)***
Treatment naïve/no anti-cancer treatment	-	-	*11/23 (48%)*
Chemotherapy*	*2/26 (8%)*	*2/6 (33%)*	*2/23 (9%)*
Targeted therapies*	*8/26 (31%)*	*1/6 (17%)*	-
Single agent mAb*	*1/26 (4%)*	*1/6 (17%)*	-
Chemo/targeted therapies + immunotherapy*	*13/26 (50%)*	*1/6 (17%)*	*1/23 (4%)*
Lenalidomide	*1/26 (4%)*	*1/6 (17%)*	*2/23 (9%)*
Immune checkpoint inhibitor	-	-	*1/23 (4%)*
Radiotherapy	*1/26 (4%)*	-	*4/23 (17%)*
Surgery		-	*1/23 (4%)*
**Following 1st dose (n=55)**	***49% (n=27)***	***51% (n=28)***	
Treatment naïve/no anti-cancer treatment		*18/28 (64%)*	
Chemotherapy*	*5/27 (19%)*	*1/28 (4%)*	
BTK inhibitors *	*6/27 (22%)*	*2/28 (7%)*	
Single agent mAb*	*1/27 (4%)*	*2/28 (7%)*	
Chemo/targeted therapies + immunotherapy*	*11/27 (41%)*	*4/28 (14%)*	

Lenalidomide	*3/27 (11%)*	-	
Immune checkpoint inhibitor		*1/28 (4%)*	
Radiotherapy	*1/27 (4%)*		
**Prior to the Day 21 2nd dose (n=6)**	***33% (n=2)***	***67%(n=4)***	
No anti-cancer treatment	-	*4/4 (100%)*	
BTK inhibitor	*2/2 (100%)*	-	
**Following the Day 21 2nd dose (n=6)**	***33% (n=2)***	***67%(n=4)***	
No anti-cancer treatment	-	*4/4 (100%)*	
BTK inhibitor	*2/2 (100%)*	-	

+ missing data solid cancers: n=3; haematological cancers: n=1.
**Chemotherapy regimens include mini-CHOP, hydroxycarbamide, mercaptopurine, methotrexate; targeted therapies include BTKi, Bcl2i, bortezomib; monoclonal antibodies include: Anti-CD30, Anti-CD20; Chemo/targeted therapies + immunotherapies include combination therapies with anti-CD20, Anti-CD30, Anti-CD38 therapies*.

Routine full blood count performed at each trial specific visit.

**Routine blood parameters**				
Timepoint	**Pre-Vaccine**	**3 weeks following one dose of BNT162b1 vaccine**	**5 weeks following one dose of BNT162b1 vaccine**	**At 5 weeks following D1 & D21 dosing**
**Solid Cancers**				
Total numbers	75	65	21	19
Hb	121 (79-175)	119 (15-152)	113 (93-135)	121 (81-149)
WCC	6.5 (3-19)	6.3 (2.1-25)	4.3 (1.1-18)	6.3 (3.8-11.6)
Platelets	245 (90-688)	231 (195-668)	210 (52-989)	276 (122-411)
Neutrophils	4 (1.2-16)	4.2 (1-22.5)	2.6 (0.7-15.3)	4.6 (1.9-7.8)
Lymphocytes	1.5 (1.2-16)	1.4 (0.4-5)	1.1 (0.4-3.2)	1.5 (0.7-4.7)
Basophils	0.1 (0-1.5)	0.1 (0-0.4)	0.1 (0-0.2)	0.1 (0-0.2)
Eosinophils	0.1 (0-0.8)	0.1 (0-1.9)	0.2 (0-0.5)	0.1 (0-0.2)
Monocytes	0.8 (0-1.5)	0.6 (0.1-1.9)	0.7 (0-1.6)	0.6 (0.3-1)
CRP	14.2 (0-78)	4 (1-149)	3 (1-111)	2 (1-45)
**Haematological Cancers**				
Total numbers	38	34	8	2
Hb	127 (70-127)	119 (74-171)	116.5 (95-146)	N/A
WCC	5.2 (1-101)	4.6 (0.5-51.7)	3.6 (1.1-18)	
Platelets	185 (13-473)	166 (51-514)	145 (52-276)	
Neutrophils	5.5 (0.1-13.1)	2.7 (0.4-22.9)	2.1(0.7-15.3)	
Lymphocytes	1.6 (0.2-97)	1.05 (0.1-179)	0.8 (0.4-3.2)	
Basophils	0.1 (0-0.9)	0.1 (0-0.3)	0.1 (0-0.1)	
Eosinophils	0.1 (0-0.5)	0.1 (0-0.7)	0.2 (0-0.3)	
Monocytes	0.8 (0.1-2.5)	0.5 (0-2.1)	0.5 (0-1.6)	
CRP	2 (1-144)	3 (1-75)	1.5 (1-15)	

Serological and T cell responses at week 3 following single inoculum of the vaccine stratified by cancer subtypes

	Serological response TP2	T cell response TP2
**Solid Malignancies**	**21/56**	**22/31**
Women's Cancers (Gynae, breast)	9/19 (47%)	9/10 (90%)
Urological Cancers (Renal, Prostate, testicular, bladder)	4/6 (67%)	4/4 (100%)
Skin Cancers (Melanoma, Merkel cell)	2/9 (22%)	4/7 (57%)
Thoracic malignancies (Lung, Mesothelioma)	3/14 (21%)	2/6 (33%)
GI Cancers (Stomach, oesophageal, pancreas, CRC)	3/6 (50%)	3/4 (75%)
Others	0/2 (0%)	
**Haematological malignancies**	**8/44**	**9/18**
Mature B -cell neoplasms	6/31	7/13
Chronic lymphocytic leukaemia/Small lymphocytic lymphoma	1/6 (16%)	2/5 (40%)
Plasma Cell Myeloma	3/9 (33%)	2/3 (66%)
Diffuse large B cell lymphoma	1/6 (16%)	-
Follicular lymphoma	1/4 (25%)	-
Lymphoplasmacytic lymphoma	0/1 (0%)	1/1 (100%)
Burkitt's lymphoma	0/1 (0%)	1/1 (100%)
Mantle cell Lymphoma	0/1 (0%)	-
MALT lymphoma	0/1 (0%)	1/1 (100%)
Nodular sclerosing Hodgkin lymphoma	0/1 (0%)	0/1 0%)
Post-renal transplant lympho-proliferative disorder	0/1 (0%)	0/1 (0%)
Mature T cell neoplasms	1/4	1/1
Anaplastic large cell lymphoma	1/4 (1%)	1/1 (1%)
Myeloid and acute leukaemia neoplasm	1/8	1/4
Acute myeloid leukaemia	0/1 (0%)	-
Myelodysplastic Syndrome/Myeloproliferative Neoplasm (MDS/MPN)	1/2 (50%)	0/1 (0%)
Chronic myelomonocytic leukaemia (CMML)	0/2 (0%)	0/1(0%)
T-cell precursor acute lymphoblastic leukaemia	0/2(0%)	0/1 (0%)
Myelofibrosis	0/1 (0%)	1/1 (0%)
Others	0/1	
Amyloid light-chain (AL) amyloidosis	0/1 (0%)	

Serological and T cell responses at week 5 following first dose of the COVID-19 vaccine stratified by cancer subtypes

	No boost		D21 boost	
	**Serological response**	**T cell response**	**Serological response**	**T cell response**
**Solid Malignancies**	**10/33**	**8/15**	**18/19**	**14/16**
Women's Cancers (Gynae, breast)	3/12 (25%)	4/6 (66%)	6/6 (100%)	4/4 (100%)
Urological Cancers (Renal, Prostate, testicular, bladder)	4/7 (57%)	3/4 (75%)	1/1 (100%)	
Skin Cancers (Melanoma, Merkel cell)	2/3 (66%)	1/2 (50%)	4/5 (80%)	5/5 (100%)
Thoracic malignancies (Lung, Mesothelioma)	1/7 (14%)	0/3 (0%)	3/3 (100%)	3/3 (100%)
GI Cancers (Stomach, oesophageal, pancreas, CRC)	0/2 (0%)		4/4 (100%)	2/4 (50%)
Others	0/2 (0%)			
**Haematological malignancies**	**4/36**	**6/18**	**3/5**	**3/4**
Mature B -cell neoplasms	2/24	3/13	1/4	2/3
Chronic lymphocytic leukaemia/Small lymphocytic lymphoma	0/6 (0%)	1/4 (25%)	1/2 (50%)	1/1 (100%)
Plasma Cell Myeloma	1/7 (14%)	1/4 (25%)	1/1 (100%)	1/1 (100%)
Diffuse large B cell lymphoma	0/2 0%)	0/1 (0%)		
Follicular lymphoma	1/4 (25%)			
Lymphoplasmacytic lymphoma			0/1 (0%)	0/1 (0%)
Burkitt's lymphoma	0/1 (0%)	0/1 (0%)		
Mantle cell Lymphoma	0/1 (0%)			
MALT lymphoma	0/1 (0%)	1/1 (100%)		
Nodular sclerosing Hodgkin lymphoma	0/1 (0%)	0/1 (0%)		
Post-transplant lympho-proliferative disorder	0/1 (0%)	0/1 (0%)		
Mature T cell neoplasms	1/3	1/1		
Anaplastic large cell lymphoma	1/3 (47%)	1/1 (47%)		
Angioimmunoblastic T-cell lymphoma				
Myeloid and acute leukaemia neoplasm	1/7	2/4	1/1	1/1
Acute myeloid leukaemia	0/1 (0%)			
Myelodysplastic Syndrome/Myeloproliferative Neoplasm (MDS/MPN)	1/1 (100%)		1/1 (100%)	1/1 (100%)
Chronic myelomonocytic leukaemia (CMML)	0/2 (0%)	0/1 (0%)		

T-cell precursor acute lymphoblastic leukaemia	0/2 (0%)	1/2 (50%)		
Myelofibrosis	0/1 (0%)	1/1 (100%)		
Others	0/2			
Osteomyelofibrosis	0/1 (0%)			
Amyloid light-chain (AL) amyloidosis	0/1 (0%)			

List of antibodies for the antibody staining mix for flow cytometry

Company	Catalog number	Description	Format	Dilution
Biolegend	318310	CD56	APC	1:50
Biolegend	325610	CD14	AF488	1:100
Biolegend	304026	CD45	PerCP	1:200
Biolegend	317342	CD3	APC-Cy7	1:200
Biolegend	302208	CD19	PE	1:200
Biolegend	302016	CD16	PE-Cy7	1:800
Biolegend	323032	CD15	BV605	1:400
Biolegend	312226	CD10	BV711	1:40
Biolegend	305020	CD64	BV421	1:40
Biolegend	304830	CD62L	BV785	1:100
Biolegend	302240	CD19	BV786	1:100
Biolegend	301830	CD14	BV421	1:100
Biolegend	354208	CD303	FITC	1:100
Biolegend	305406	CD86	PE	1:100
BD	563083	HLA-DR	BV510	1:100
Biolegend	331524	CD1c	APC	1:100
Biolegend	301638	CD11c	BV650	1:100
Biolegend	306016	CD123	PerCP-Cy5.5	1:100
Biolegend	317340	CD3	AF700	1:200
Biolegend	302016	CD16	PE-Cy7	1:200

Sensitivity and specificity for each individual response using a threshold of ≥7 cytokine secreting cells/10^6.

Response	Area under the ROC curve	Sensitivity	Specificity
IFN-γ to RBD	0.831	56.3%	100%
IL-2 to RBD	0.824	62.5%	100%
IFN-γ to S2	0.790	62.5%	76.5%
IL-2 to S2	0.899	81.3%	100%

Appendix Figure 1: Trial design and enrolment

Schematic to show the time points at which trial activities (blood tests, nasal swab samples and safety checks) were performed in relation to the vaccination doses.
Time-point 1 (TP1) = pre vaccine, TP 2 = 3 weeks following 1st dose and TP3 = 5 weeks following 1st dose.

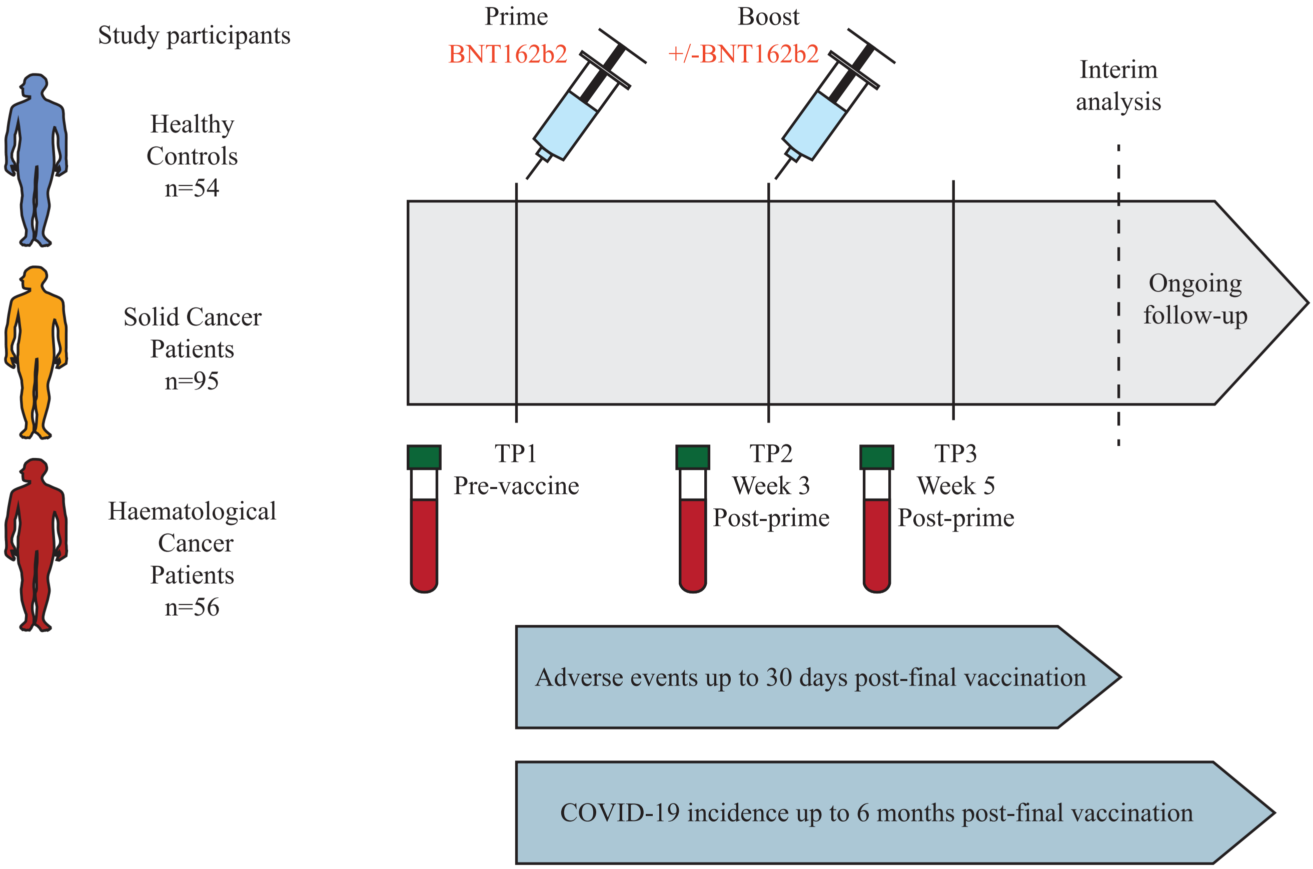

Appendix Figure 2. Natural infection and neutralisation responses following BNT162b2 vaccination.

A. Incidence of PCR swab-confirmed SARS-CoV-2 infection within patients and healthy controls following first dose of vaccine.

B. Progression of PCR swab-confirmed SARS-CoV-2 infection following first dose of vaccine.

C. Spike-specific IgG titres prior to vaccination in healthy controls (n=14), solid tumour cancer patients (n=64) and haematological cancer patients (n=38), illustrating seropositivity threshold (horizontal line at EC50 dilution of 70) and suspected pre-exposed individuals.

D. N-specific IgG titres prior to and following vaccination, highlighting PCR-swab confirmed and baseline Spike-high healthy controls (n=4) and solid tumour cancer patients (n=5) (red), and other healthy controls (n=2), solid tumour cancer patients (n=4) and haematological cancer patients (n=5) suspected of pre-existing or developing SARS-CoV-2 infection due to high N IgG titres (grey). Horizontal line indicates N-specific IgG threshold for suspected infection. White dots indicate remaining healthy controls (n=42), solid tumour cancer patients (n=63) and haematological cancer patients (n=46) from combined vaccination cohorts.

E. Age distribution of healthy controls (n=34), solid tumour cancer patients (n=56) and haematological cancer patients (n=44) when grouped by Spike-specific IgG titres into above threshold (>70), below threshold (25-70) and below limit of detection (<25) at 3 weeks post-vaccination.

F. Neutralization titres against WT and B.1.1.7 strains SARS-CoV-2 from sera of healthy controls (n=16) at 3 weeks post-vaccination. Paired Wilcoxon test.

G. Baseline T-cell and B-cell counts/ml of blood in non-responders (NR) and responders (R) segregated by cancer type; solid (yellow) n=31 (R=14/NR=17) and Haematological (red) n=23 (R=4/NR=19). Short bars represent median values for NR and R. Wilcoxon test corrected by Benjamini-Hochberg method; no significant differences between groups.

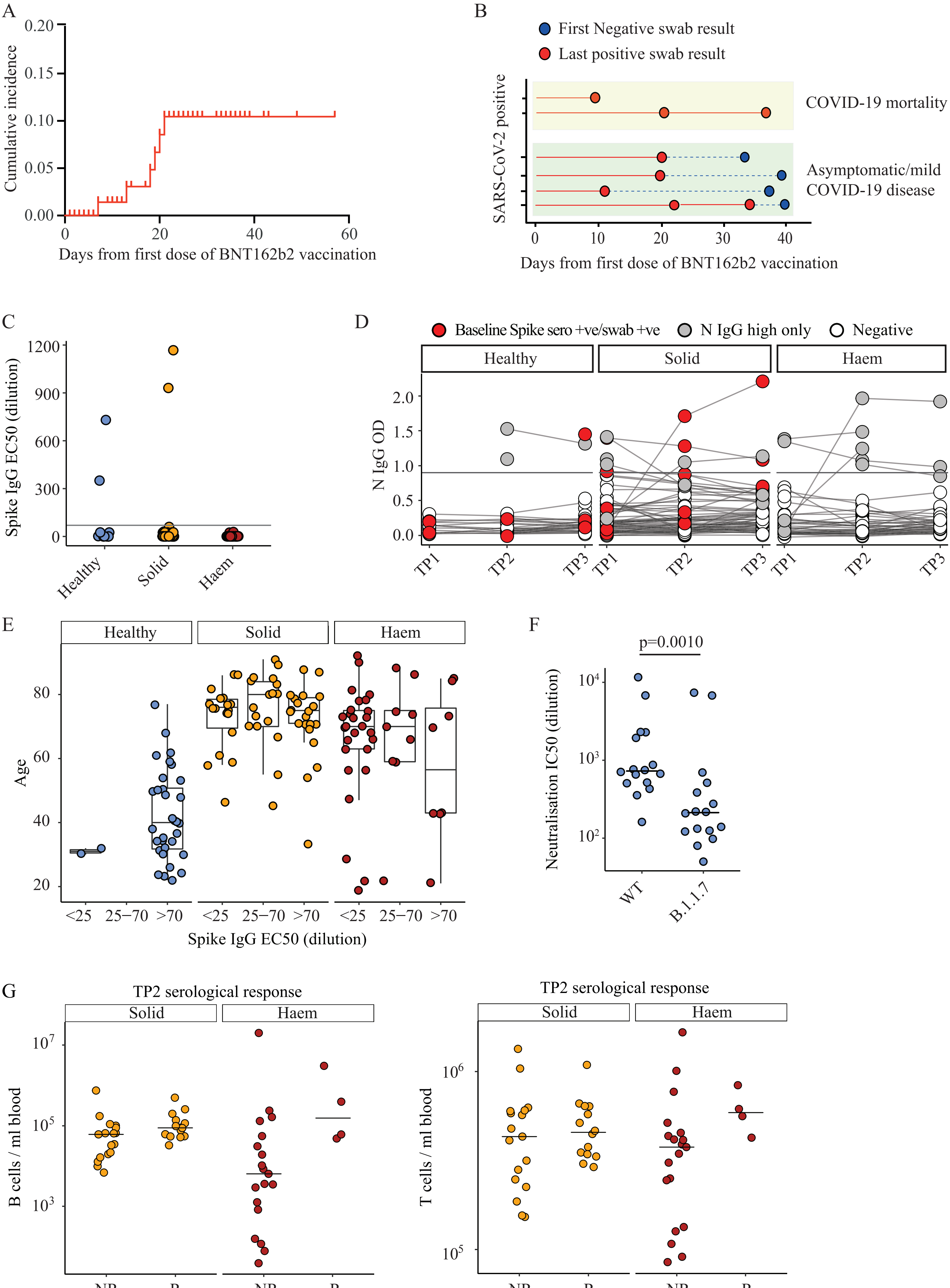
A
Cumulative incidence
Days from first dose of BNT162b2 vaccination
B
First Negative swab result
Last positive swab result
SARS-CoV-2 positive
COVID-19 mortality
Asymptomatic/mild COVID-19 disease
Days from first dose of BNT162b2 vaccination
C
Spike IgG EC50 (dilution)
Healthy
Solid
Haem
D
Baseline Spike sero +ve/swab +ve
N IgG high only
Negative
Healthy
Solid
Haem
N IgG OD
TP1
TP2
TP3
E
Healthy
Solid
Haem
Age
<25
25−70
>70
Spike IgG EC50 (dilution)
F
p=0.0010
Neutralisation IC50 (dilution)
WT
B.1.1.7
G
TP2 serological response
Solid
Haem
B cells / ml blood
NR
R
TP2 serological response
Solid
Haem
T cells / ml blood
NR
R

Appendix Figure 3. QC and combined T-cell responses to COVID-19 vaccine BNT162b2

A. IFN-γ^+ and IL-2^+ response to stimulation with peptides from RBD and S2 was reported as number of spots per 10^6 cells in a negative and a positive PBMC sample that was run alongside for every assay. Variation in the response among different batches was assessed with CVs as follows: IFN-γ in S2 (1.702, 0.198), IFN-γ in RBD (1.380, 0.155), IL-2 in S2 (0.943, 0.222), IL-2 in RBD (1.133, 0.209) for the negative and the positive controls, respectively.

B. Determining a cut-off value for assigning positive and negative fluorospot responses. Graph represents a ROC plot showing assay diagnostic sensitivity against specificity (one minus proportion of false positives) following detection of IFN-γ and IL-2 fluorospot responses to RBD and S2 peptide pools in healthy control subjects before and following one dose of BNT162b2. By convention, we selected the cut-off value that provides an operating position nearest to that of the "perfect test" (i.e., closest approximation to 100% sensitivity and 100% specificity), which was ≥7 cytokine secreting cells/10^6 PBMC.

C. Exemplary Fluorospot images for PBMC taken at TP2 (21days after 1st inoculation). IFN-γ-secreting cells are shown in green, IL-2 secreting cells in magenta and cells co-secreting both cytokines are visualised as white.

D. IFN-γ^+ and IL-2^+ dual producers after stimulation with peptides from RBD, S2 and CEF-CEFT was reported as number of spots per 10^6 cells in PBMC samples at 3weeks post vaccine in healthy controls n=17 (blue), solid tumour cancer patients n=31 (yellow) or haematological cancer patients n=18 (red). Bar represents median value by group, horizontal line represents T cell response threshold (7). Kruskal-Wallis test with Dunn's post-hoc test, corrected by Benjamini-Hochberg method.

E. Composition of i) solid tumour cancer patient and ii) haematological cancer patient cohorts tested for serology (ELISA; solid n=56, haem n=44) and T cell responses (fluorospot; solid n=31, haem n=18) at 3 weeks post-vaccination, in terms of cancer types and treatment given within 15 days either side of vaccination. Missing treatment information: solid cancers: n=3; haematological cancers: n=1.

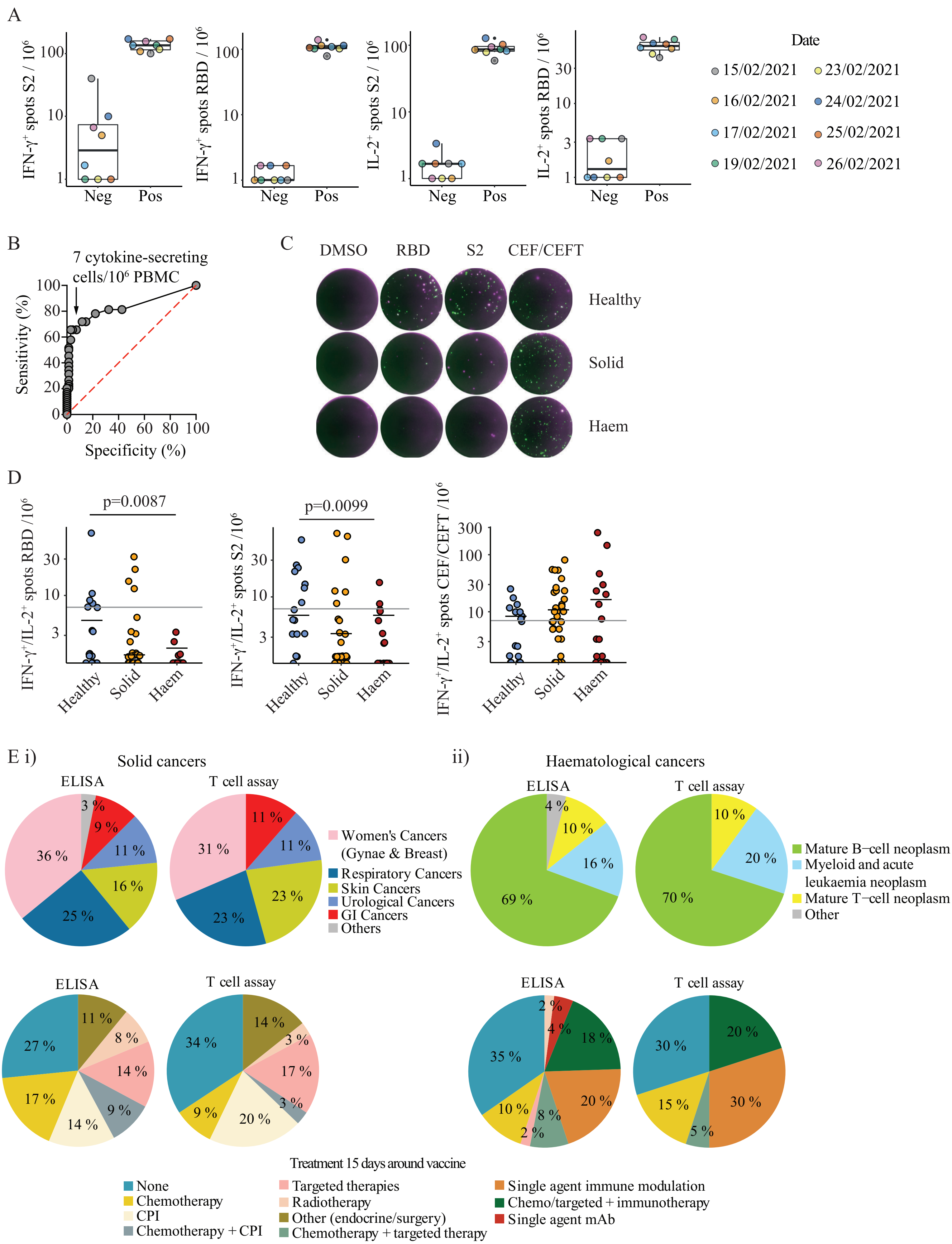

A
IFN-γ+ spots S2 / 10^6
IFN-γ+ spots RBD / 10^6
IL-2+ spots S2 / 10^6
IL-2+ spots RBD / 10^6
Neg
Pos
Date
15/02/2021
16/02/2021
17/02/2021
19/02/2021
23/02/2021
24/02/2021
25/02/2021
26/02/2021
B
7 cytokine-secreting cells/10^6 PBMC
Sensitivity (%)
Specificity (%)
C
DMSO
RBD
S2
CEF/CEFT
Healthy
Solid
Haem
D
p=0.0087
p=0.0099
IFN-γ+/IL-2+ spots RBD /10^6
IFN-γ+/IL-2+ spots S2 /10^6
IFN-γ+/IL-2+ spots CEF/CEFT /10^6
Healthy
Solid
Haem
E i)
Solid cancers
ELISA
T cell assay
Women's Cancers (Gynae & Breast)
Respiratory Cancers
Skin Cancers
Urological Cancers
GI Cancers
Others
ii)
Haematological cancers
Mature B−cell neoplasm
Myeloid and acute leukaemia neoplasm
Mature T−cell neoplasm
Other
Treatment 15 days around vaccine
None
Chemotherapy
CPI
Chemotherapy + CPI
Targeted therapies
Radiotherapy
Other (endocrine/surgery)
Chemotherapy + targeted therapy
Single agent immune modulation
Chemo/targeted + immunotherapy
Single agent mAb

Figure S3

Appendix Figure 4. Impact of disease and treatments on serological response.

A. The number of serological responders and non-responders were assessed across haematological cancer patients at TP2 (n=43), and segregated by the type of cancer.

B. The number of serological responders and non-responders were assessed across solid cancer patients at TP2 (n=54), and segregated by the type of cancer.

C. The number of serological responders and non-responders were assessed across solid cancer patients at TP2 (n=43), and segregated based on the type of treatment received within 15 days either side of first vaccination.

D. The number of serological responders and non-responders was assessed across haematological cancer patients at TP2 (n=44), and segregated based on steroid treatment within 15 days either side of first vaccination.

E. The number of serological responders and non-responders was assessed across solid cancer patients at TP2 (n=56), and segregated based on steroid treatment within 15 days either side of first vaccination.

F. The number of serological responders and non-responders was assessed across solid cancer patients at TP2 (n=15), and segregated based on treatment with chemotherapy alone or in combination with high dose dexamethasone within 15 days either side of first vaccination..

A-C) Chi Squared test. **D-F)** Fisher's exact test.

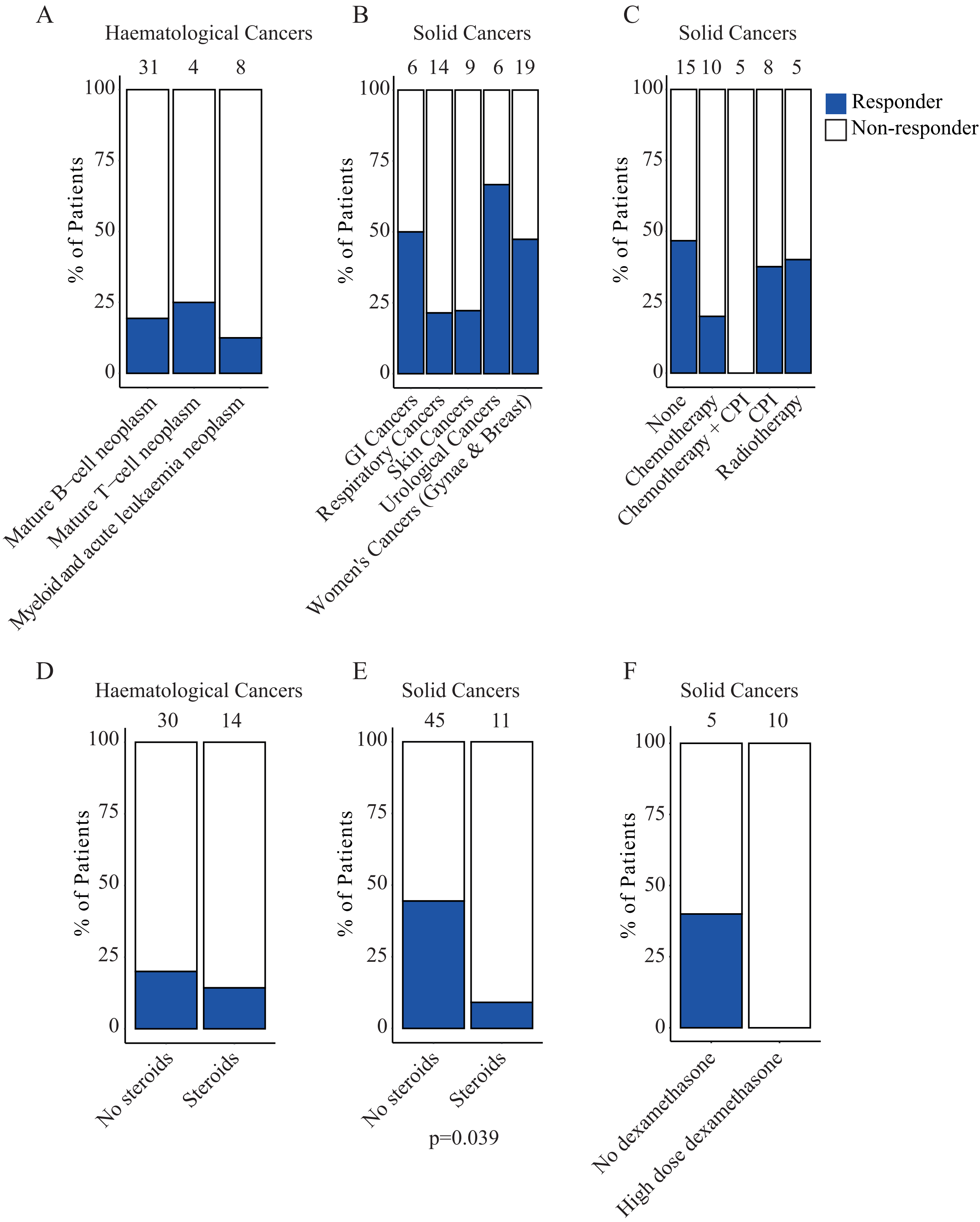
A
Haematological Cancers
31 4 8
% of Patients
0 25 50 75 100
Mature B−cell neoplasm
Mature T−cell neoplasm
Myeloid and acute leukaemia neoplasm
B
Solid Cancers
6 14 9 6 19
GI Cancers
Respiratory Cancers
Skin Cancers
Urological Cancers
Women's Cancers (Gynae & Breast)
C
Solid Cancers
15 10 5 8 5
None
Chemotherapy
Chemotherapy + CPI
CPI
Radiotherapy
Responder
Non-responder
D
Haematological Cancers
30 14
No steroids
Steroids
E
Solid Cancers
45 11
No steroids
Steroids
p=0.039
F
Solid Cancers
5 10
No dexamethasone
High dose dexamethasone

Figure S4

Appendix Figure 5: Serological and T cell responses in vaccinated individuals with concomitant SARS-CoV-2 infection.

Patients returning a positive SARS-CoV-2 PCR swab and those displaying baseline seropositivity to Spike or high N-specific IgG titres at any point (all red; PCR swab positive also diamond) were excluded from the prior analysis. Here they are shown in the context of other patients who were not suspected of natural infection (white). Lines link repeated samples from individual patients.

A. Spike-specific IgG titres in suspected naturally infected healthy controls (n=6), solid tumour cancer patients (n=9), haematological cancer patients (n=5), as compared to the remaining cohort of healthy controls (n=42), solid tumour cancer patients (n=63) and haematological cancer patients (n=46). Horizontal line indicates Spike seropositivity threshold (70).

B. IFN-γ^+ T cell response to S2 peptides in suspected naturally infected healthy controls (n=3), solid tumour cancer patients (n=4), haematological cancer patients (n=1), as compared to the remaining cohort of healthy controls (n=17), solid tumour cancer patients (n=31) and haematological cancer patients (n=22). Horizontal line indicates T cell response threshold (7).

C. IL-2$^+$ T cell response to S2 peptides in suspected naturally infected healthy controls (n=3), solid tumour cancer patients (n=4), haematological cancer patients (n=1), as compared to the remaining cohort of healthy controls (n=17), solid tumour cancer patients (n=31) and haematological cancer patients (n=22). Horizontal line indicates T cell response threshold (7).

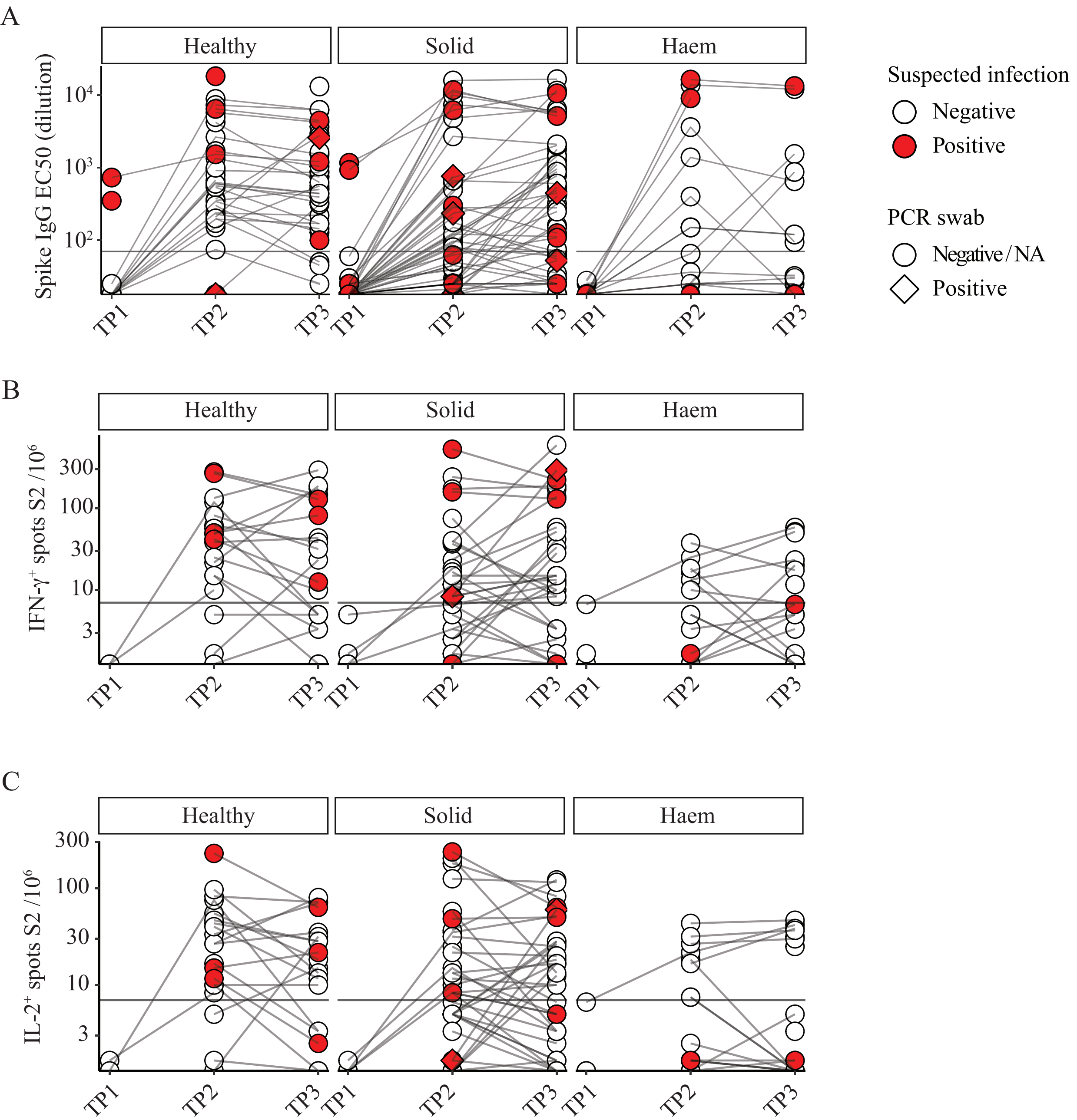

A
Healthy
Solid
Haem
Spike IgG EC50 (dilution)
10^4
10^3
10^2
TP1
TP2
TP3
Suspected infection
Negative
Positive
PCR swab
Negative/NA
Positive
B
IFN-γ$^+$ spots S2 /10^6
300
100
30
10
3
C
IL-2$^+$ spots S2 /10^6

Figure S5

FS protocol for COVID-19 projects

Peter Gorer Department of Immunobiology, School of Immunology and Microbial Sciences,
King's College London, London, UK

Day 1 – Coating FS plates

1. Remove FS plates from the package and label them as follows:

		1	2	3	4	5	6	7	8	9	10	11	12
	**A**	neg	neg	neg	RBD	RBD	RBD	SII	SII	SII	+	+	+
Pos control	**B**	neg	neg	neg	RBD	RBD	RBD	SII	SII	SII	+	+	+
Neg control	**C**	neg	neg	neg	RBD	RBD	RBD	SII	SII	SII	+	+	+
	**D**	neg	neg	neg	RBD	RBD	RBD	SII	SII	SII	+	+	+
	**E**	neg	neg	neg	RBD	RBD	RBD	SII	SII	SII	+	+	+
	**F**	neg	neg	neg	RBD	RBD	RBD	SII	SII	SII	+	+	+
	**G**	neg	neg	neg	RBD	RBD	RBD	SII	SII	SII	+	+	+
	**H**	neg	neg	neg	RBD	RBD	RBD	SII	SII	SII	+	+	+

Dose 1 – Follow-up – Controls →

	1	2	3	4	5	6	7	8	9	10	11	12
**A**	neg	neg	neg	RBD	RBD	RBD	SII	SII	SII	+	+	+
**B**	neg	neg	neg	RBD	RBD	RBD	SII	SII	SII	+	+	+
**C**	neg	neg	neg	RBD	RBD	RBD	SII	SII	SII	+	+	+
**D**	neg	neg	neg	RBD	RBD	RBD	SII	SII	SII	+	+	+
**E**	neg	neg	neg	RBD	RBD	RBD	SII	SII	SII	+	+	+
**F**	neg	neg	neg	RBD	RBD	RBD	SII	SII	SII	+	+	+
**G**	neg	neg	neg	RBD	RBD	RBD	SII	SII	SII	+	+	+
**H**	neg	neg	neg	RBD	RBD	RBD	SII	SII	SII	+	+	+

Conditions:

1 Combo (2 plates): 24 wells/condition x 2 x 75ul = 3.6 ml 4 ml

2 Combos (4 plates): 24 wells x 4 x 75ul = 7.2 ml **6, 7** OR **8 ml**

FS antibodies:

96 wells/plate x 2 plates (1 combo) = 1 wells x 100 ul 19.2 ml 20 ml (excess)

2. Dilute capture antibodies in sterile 1x PBS as specified:
   Total volume = **20 ml**

   (A) Triple 1 (22-24h stimulation): **IFNg/IL-2/Granzyme B**

Anti-IFNg	Anti-IL-2	Anti-Granzyme B
1 mg/ml 15 µg/ml<br>300 µl	0.5 mg/ml 15 µg/ml<br>600 µl	1 mg/ml 15 µg/ml<br>300 µl

   (B) Triple 2 (46-48h stimulation): **IFNg/IL-22/IL-17A**

Anti-IFNg	Anti-IL-22	Anti-IL-17A
1 mg/ml 15 µg/ml<br>300 µl	0.5 mg/ml 15 µg/ml<br>600 µl	0.5 mg/ml 15 µg/ml<br>600 µl

3. Pre-wet the membrane of the FluoroSpot plates with 35% EtOH, 15 µl/well for a maximum of 1 min (use an electronic 12 channel pipette) 3.5 ml EtOH + 6.5 ml sterile water (autoclaved) (prepare 2 in 30 ml universal tubes) Discard EtOH after 10-15 seconds of last row added and promptly start washes with water
   https://www.mabtech.com/knowledge-center/tutorials-and-guidelines/etoh-treatment-elispot-plates
4. Wash the plate 4 times with sterile water, 200 µl/well (use an electronic 12 channel pipette)
5. Add capture antibody solution (use a manual multichannel pipette), 100 µl/well and incubate overnight at 4–8 °C (add plastic to avoid evaporation)

Day 2 – Thawing, counting and cell preparation

Preparation of reagents
(enough for 4 plates)

(1) **Complete media** (RPMI + PSF) RPMI in the cold room, PSF aliquots in the -20C in the main lab (10 ml aliquots in 15 ml falcon tubes). **Prepare 1 each day**

(2) **Thawing media** (complete media + 10% FBS) In 50 ml falcon tubes, filter and place in the waterbath
(**6** FBS in 15 ml falcon tubes)

(3) **10% sigma AB media** (complete media + 10% sigma AB) In 50 ml falcon tubes, filter and place **x3** in the waterbath and **x2** at RT inside the hood
(**5** bijous with Sigma AB serum **#322**)

1. Label 15 ml falcon tubes with numbers (1-8) and (9-16)
2. Prepare Pasteur pipettes, cryovial rack and Cool cell box (purple box) with dry ice and metal rack for the thawing step
3. Double check sample location before taking cryovials out of the Liquid Nitrogen (LN)
4. Remove cryovials from the LN and place them on dry ice. Remember to take 1 aliquot of neg control and 2 of positive control
5. Check samples are in the right order and place them into the water bath using the cryovial rack
6. Shake them constantly until a small ice lump is left
7. Top up the vial dropwise with thawing media using a Pasteur pipette
8. Transfer contents of the cryovials into a 15 ml falcon
9. Top up the tube to 10 ml with thawing media and centrifuge cells at 400x*g* for 5 min at RT
10. Discard supernatant and resuspend cells in 10 ml of thawing media
11. Place them at 37ºC in the incubator for 1 hour (remember to uncap the 15 ml falcons slightly to allow exchange of gases)
12. After resting cells for 1 hour, centrifuge cells at 400x*g* for 5 min at RT (to eliminate cell debris)
13. Discard supernatant and resuspend cells in **x ml** of thawing media (to have 2x10^6 cells/ml) and record resuspension volume in the ***#FS_COVIDIP2_Cell count worksheet***
14. Count cells in the haemocytometer (duplicates) and calculate volume of 10% Sigma AB media required to have **2.67x10^6 cells/ml** (200.000 cells/75 µl) using ***#FS_COVIDIP2_Cell count worksheet***
15. Spin again at 400x*g* for 5 min at RT

16. Resuspend cells in the according volume of 10% Sigma AB media or in 1.5 ml if volume is too high to run through FACS tubes with cell strainer snap cap
17. Run cells through FACS tubes with cell strainer snap cap (to eliminate cell clumps) and if volume is too high, transfer back to either 15 ml or 50 ml
18. Add necessary volume of 10% Sigma AB media and cap the tubes with white FACS tube caps (to keep them sterile)
19. Flick off media from 96-well FS plate (from blocking step) and add 75 µl of cells into each well (using a one channel pipette). For this step, pipette up and down with a P1000 (or invert tube) 3-4 times before taking 75 µl of cells (do not pump cells up and down between wells)
20. Prepare stimuli (neg, RBD, SII or pos) into **3** 15 ml falcon tubes using pre-warmed 10% Sigma AB media. Mix properly before adding 75 µl into each well (use a manual multichannel pipette and a 25 ml reservoir). Remember to change tips every time
21. Incubate plate at 37°C in the incubator for 22-24h (Triple 1, **A**) **OR** 46-48h (Triple 2, **B**)

**Reagent**	**Storage °C**	**Aliquot volume (µl)**	**Stock concentration**	**Working concentration**	**Volume needed (µl) for 1:1000**	**Volume of media needed (ml)**
**DMSO (-)**	-20	32	na	1:2000	6, 7 or 8	6, 7 or 8
**CEF/CEFT (+)**	-20	16 + 16	500 µg/ml	1:2000	6, 7 or 8	6, 7 or 8
**RBD pool**	-20	32	500 µg/ml	1:2000	6, 7 or 8	6, 7 or 8
**SII**	-80	1.3 or 2.6	500 µg/ml	1:2000	6, 7 or 8	6, 7 or 8

Day 2 – Block FluoroSpot plates

1. Take FS plates out of the fridge and flick off capture antibody solution
2. Wash plates 5 times with sterile cold 1x PBS *During rest/1h incubation at 37ºC*
3. Add 200 µl of 10% Sigma AB media into each well (minimum 30 min at RT, no max). Use **x2** falcon tubes of 10% Sigma AB media needed (keep at RT inside the set up hood)

Day 3/4 – Detection of spots

1. Prepare **x2** fresh PBS containing 0.1 % BSA into a 50 ml falcon by adding:
   50 ml PBS + 666 µl 7.5% BSA (stock) – **Filtered**
2. Dilute the detection antibodies in **20 ml** of PBS (containing 0.1%BSA) into a universal. Add detection antibodies and vortex properly:

   (A) Triple 1 (22-24h stimulation): **IFNg/IL-2/Granzyme B**

Anti-IFNg-BAM	Anti-IL-2-biotinylated	Anti-Granzyme B-WASP
1:200<br>100 µl	0.5 mg/ml 1 ug/ml<br>(1:500) 40 µl	1:200<br>100 µl

   (B) Triple 2 (46-48h stimulation): **IFNg/IL-22/IL-17A**

Anti-IFNg-BAM	Anti-IL-22-biotinylated	Anti-IL-17A-WASP
1:200<br>100 µl	0.5mg/ml 0.5ug/ml<br>(1/1000) 20 µl	1:200<br>100 µl

3. Take out the FS plates from the incubator and transfer 30 µl of supernatant into a labelled 96-well u-bottom plate. Add a plastic cover to avoid evaporation and place in the Hayday -80ºC
4. Flick off cells and wash plates 4 times with RT 1x PBS (in bench, using an electronic 12 channel pipette). Dispense 200 µl into each well
5. After the last wash, decant the PBS from the plates by flicking until there are no droplets (do not touch the paper). Using a manual multichannel pipette, add 100 µl/well. Incubate for 2h at RT (wrapping it with foil to protect it from light). Place FS plates under a cover and check the temperature of the room (needs to be between 20-25ºC)

6. Dilute the fluorophore-conjugates in **20 ml** of PBS (containing 0.1%BSA) into a universal as specified:

   (A) Triple 1 (20h stimulation): **IFNg/IL-2/Granzyme B**

Anti-BAM-490	SA-550	Anti-WASP
1:200 100 µl	1:200 100 µl	1:200 100 µl

   (B) Triple 2 (48h stimulation): **IFNg/IL-22/IL-17A**

Anti-BAM-490	SA-550	Anti-WASP
1:200 100 µl	1:200 100 µl	1:200 100 µl

7. Flick of detection antibodies. Wash 4 times with RT 1x PBS (in bench, using an electronic 12 channel pipette) and dispense 200 µl into each well
8. After the last wash, decant the PBS from the plates by flicking until there are no droplets (do not use paper). Using a manual multichannel pipette, add 100 µl/well. Incubate for 1h at RT (wrapping it with foil to protect it from light). Place FS plates under a cover and check the temperature of the room (needs to be between 20-25ºC)
9. Flick of fluorophore-conjugates and wash 4 times with RT 1x PBS (in bench, using an electronic 12 channel pipette) and dispense 200 µl into each well
10. Decant the PBS from the plates by flicking until there are no droplets. Using a manual multichannel pipette, add 50 µl of Fluorescence enhancer in each well and leave the plate for 10 min at RT (wrapping it with foil to protect it from light)
11. Empty the plate and remove any residual Fluorescence enhancer
12. Carefully remove underdrain and leave the plate in the dark to dry

Day 4/5 – Counting the spots in the AID Spectrum reader

1. Plate should be completely dry before analysis. Inspect and count spots in a Fluorescence reader using the settings below:

2. Once read, store the plate wrapped in foil in dark at RT.

Experimental timeline:

Day 1: Coat FS plates (anytime)

Day 2: Block FS plates (during 1h incubation/rest)
Set up, thaw, count and incubate cells with stimuli

Day 3: Detection of spots **Triple 1** (IFNg/IL-2/GZB)

Day 4: Detection of spots **Triple 2** (IFNg/IL-22/IL-17A)
Read and count **Triple 1** (IFNg/IL-2/GZB)

Day 5: Read and count **Triple 2** (IFNg/IL-22/IL-17A)
Export files and add data into excel master file

The SOAP Study – Participant recruitment summary

Site Name	Chief investigator/Principle Investigator	No. of participants recruited
Guy's & ST Thomas NHS Foundation Trust	Dr Sheeba Irshad – CI	192
Kings College Hospital NHS Trust, Denmark Hill	Dr Piers Patten - PI	6
Princess Royal University Hospital	Dr Piers Patten - PI	7

Immunological consequences of COVID-19 for cancer patients

***SOAP** Study- **S**ars-cov-2 f**O**r c**A**ncer **P**atients*

Version number 3.0

Final/draft - Final

Date – 23rd July 2020

IRAS ID: 282337

REC ID: 20/HRA/2031

1 Introduction

As of March 24, 2020, coronavirus disease 2019 (COVID-19) has been confirmed in 125 million people worldwide, carrying a mortality of approximately 4.45%, compared with a mortality rate of less than 1% from influenza. The symptoms of COVID-19 vary from modest, mild to acute respiratory distress syndrome (ARDS), and the latter of which is generally associated with deregulated immune cytokine production; however, we currently know little as to the interplay between the extent of clinical symptoms and the compositions of the immune responses. Some patient populations (e.g. the >70yrs, patients with co-morbidities are prone to develop more severe symptoms and require emergent medical interventions. Predictors of fatality from a recent retrospective study, included elevated ferritin (mean 1297·6 ng/ml in non-survivors vs 614·0 ng/ml in survivors; p<0·001) and IL-6 (p<0·0001) and patients with severe COVID-19 might have a cytokine release syndrome (CRS). Pharmacotherapy targeted against the virus holds the greatest promise when applied early in the course of the illness, but its usefulness in advanced stages may be doubtful. Similarly, use of anti-inflammatory therapy applied too early may not be necessary and could even provoke viral replication such as in the case of corticosteroids. It appears that there are two distinct but overlapping pathological subsets, the first triggered by the virus itself and the second, the host response (figure 1).

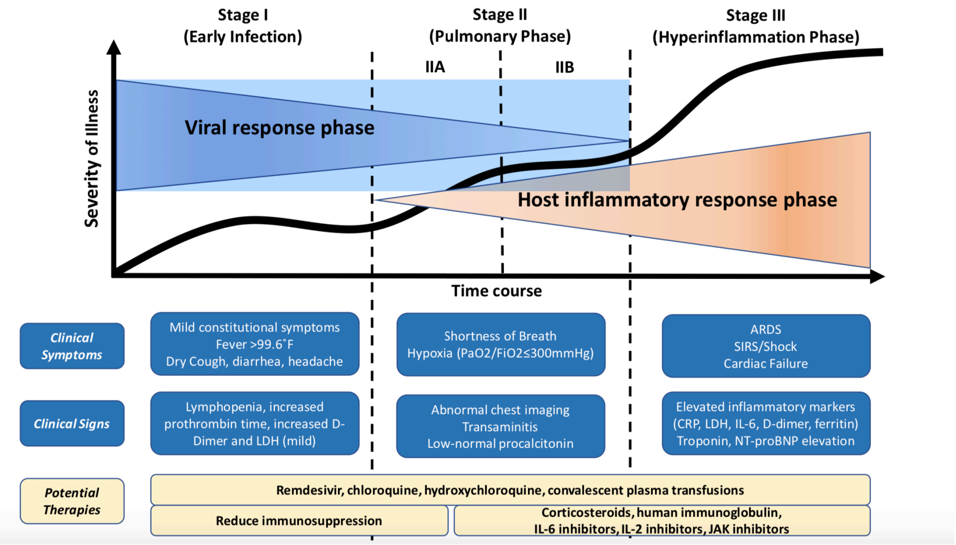

Figure 1: The figure shows 3 escalating phases of disease progression with COVID-19, with associated signs, symptoms and potential phase-specific therapies. ARDS = Acute respiratory distress syndrome; CRP = C- reactive protein; IL = Interleukin; JAK = Janus Kinase; LDH=Lactate DeHydrogenase; SIRS = Systemic inflammatory response syndrome.

The role of escape from immune surveillance mechanisms is now well recognised as a hallmark of cancer progression. Tumours evolve to prevent the activation of the immune response. The immunosuppressive effects of cancer cells are mediated by the secretion of soluble factors, by the expression of inhibitory molecules, and by turning the cellular infiltrates into tolerizing cells that can in turn suppress other potentially tumour-specific immune cells. Specific cellular immune response to SARS-CoV-2 in the context of a dysfunctional immune regulation is still largely unknown. In this study we would like to

investigate the different molecular, cellular and immunological aspects of COVID-19 related immune responses in cancer patients from varying symptomatic phases of the infection. To understand if the immune changes are specific to a COVID-19 diagnosis we would like to compare these immunological profile in COVID-19 negative cancer patients.

Furthermore, preliminary work from the SOAP study has shown that in some cancer patients, we observe delayed or negligible seroconversion, prolonged shedding, and sustained immunodysregulation. These data have implication for COVID-19 vaccine therapy in cancer patients as we need to further understand if cancer patients will mount a similar immune response to COVID-19 vaccines as non-cancer patients. The results of this study will highlight the possible need for careful oversights for cancer patients, including, for example, vaccination boosting and follow-up.

2 Trial objectives and purpose

In this sub-study we aimed to address 1) the safety and efficacy of BNT162b2 COVID-19 vaccine in cancer patients; and 2) whether or not there was a profound impact for cancer patients of boosting at day-21 post initial vaccination..

Primary endpoints
The co-primary end-point was assessment of the prevalence of humoral immunity to SARS-CoV-2 Spike (S) protein in cancer patients following the first injection of the COVID-19 mRNA BNT162b2 Vaccine, and, the impact on this of boosting after 21 days.

Secondary endpoints
The secondary endpoints of the study were: the safety following each vaccine dose, T cell responses, and neutralisation of SARS-CoV-2 Wuhan strain and of a variant of concern (VOC) (B.1.1.7). Follow-up is planned for further blood sampling after the delayed vaccine boost.

3 Study design & Flowchart

3.1 Study Design

The study is a prospective cohort study of patients with malignancy. Longitudinal blood sampling as shown in table below for those patients who have received the COVID-19 vaccine. No more than 75ml/week of blood will be taken. Every effort will be made to collect the samples at the same time as a standard of care sample is being taken for the patients' clinical care. Where possible serial nasopharyngeal SARS-CoV-2 rRT-PCR swab-tests were performed every 10-days or in cases of symptomatic COVID-19. Telephone consultations to evaluate reactogenicity and safety were scheduled weekly where possible.

3.2 Flowchart

Participant blood sampling time-points

	**Time Point 1**<br><br>Pre-Vaccine baseline bloods	**Time Point 2**<br><br>3 weeks post first vaccine bloods	**Time Point 3**<br><br>5 weeks post first vaccine bloods	**Time Point 4**<br><br>2 weeks post booster for delayed cohort
Patient information and informed consent	X			
Demographics	X			
History of symptoms		X	X	X
Blood for PBMC	X	X	X	X
Blood for Serum & Plasma	X	X	X	X

4 Subject selection

4.1 Subject inclusion criteria

Cancer Cohort

- Over 16 years of age
- Any active diagnosis of solid or haematological cancer
- Able to provide Informed consent
- Eligible for the COVID-19 vaccine

Healthy Cohort

- Over 16 years of age
- No history of an active cancer diagnosis
- Able to provide Informed consent
- Eligible for the COVID-19 vaccine

4.2 *Subject exclusion criteria*

- ≤16 years of age
- Unable to provide informed consent

5 Study procedures

5.1 *Subject recruitment*

Potential study participants may be identified based on site-specific processes. The participants will be approached by the direct care team and offered the patient information sheet. If they are interested in participation, after sufficient time to consider the study, they will meet with a member of the study team to go over questions and then informed, written, consent will be taken.

The study participant must sign and date the latest approved version of the *Informed Consent Form* before any study specific procedures are performed. Written versions of the *Participant Information Sheet* and *Informed Consent Form* will be presented to the participants detailing the rationale of the study, what it will involve for the participant; the known study specific side effects and any study specific risks involved in taking part. It will be clearly stated that the participant is free to withdraw from the study at any time for any reason without affecting their treatment and with no obligation to give the reason for withdrawal.

The participant will be allowed as much time as needed to consider the information, and the opportunity to question the investigator to decide whether they will participate in the study.

5.2 *Sample Collection/Labelling/Logging*

All samples will be allocated a study number and pseudo-anonymised. All sample handling will be carried out according to established standard operating procedures held and maintained in the Faculty of Dept of Infectious Disease Samples will be stored under the supervision of the chief investigator. Transfer of samples will be undertaken after the establishment of material transfer agreements (MTAs) and service level agreements (SLAs) as required.

Tissue storage will be undertaken under the King's College London Research Human Tissue Authority licensed. Control and quality management system policies, with regular audits, are in place.

5.3 Sample Storage Procedures

All sample handling will be carried out according to established standard operating procedures (SOPs) held and maintained in the Department of Infectious disease. Any samples collected and not tested immediately will be stored in designated HTA licensed freezers. Stored samples will be recorded in freezer logs containing the following information:

- Sample ID
- Date of sample collection
- Sample type
- Sample volume
- Date of sample received
- Sample received and logged by (staff initials).
- Location of sample

5.4 Data Recording/Reporting

The Chief Investigator will act as custodian for the study data. Data will be stored at King's College London. The following guidelines will be strictly adhered to:
Patient data will be pseudo-anonymised.

- Each patient will be allocated a unique study identification number as follows:
  - Study code: SOAP-XXX
  - Numbers will be sequential starting from 001
  - Each study sample will have a unique code, which will also be included on samples labels for storage.
- All pseudo-anonymised data will be stored on a password-protected computer.

Patient identifiable information, such as date of birth and postcode, will be held separately by the clinical research team on secure NHS computers owned and managed by the NHS foundation trust where the patient is recruited. Such information will be stored to allow later linkage for long term follow-up of patients. The patient identifiable information will be stored alongside record of the unique patient ID number assigned at point of entry into the study. Only named members of the clinical research team will have the necessary access permissions to view this personally identifiable database.

All data will be handled in accordance with the Data Protection Act 2018 and General Data Protection Guidance (2018).

5.5 Trial Steering Committee

The committee will consist of the chief investigators and all co-investigators listed on the first pages of this protocol.

The role of the committee will be oversight of data collection and sample storage and review of data generated.

The committee will meet monthly.

5.6 Ethics & Regulatory Approvals

The study will be approved by the Health Research Authority and research ethics committee (REC).

The R&D department of Guy's and St Thomas' NHS Trust is annually auditing 10% of non-CTIMP study site files at the Trust.

The CI will additionally take responsibility for delegating self-monitoring and audits onsite, and will permit REC reviews and regulatory inspections by providing direct access to source data and other study documents.

6 Compliance and withdrawal

6.1 Withdrawal / dropout of subjects

Participants have the right to withdraw from the study at any time for any reason. The investigator also has the right to withdraw patients from the study in the event of inter-current illness, protocol violations, or for administrative reasons. Should a patient decide to withdraw from the study all efforts will be made to report the reason for withdrawal as thoroughly as possible.

Participants who wish to withdraw from the study will be asked to confirm whether they are still willing to provide data collected as per routine clinical practice.

7 Financing and Insurance

7.1 Funding

Funding for the study has been secured from the KCL Charity funds awarded to Dr Irshad research group.

7.2 Insurance

KCL no fault insurance covers the design of the study. NHS bodies are legally liable for the negligent acts and omissions of their employees. Non-negligent harm is not covered by the NHS indemnity scheme. NHS indemnity operates in respect of the clinical treatment that is provided.

8 References

1. Prompetchara, E.,Ketloy, C.&Palaga, T. Immune response in COVID-19 and potential vaccines: Lessons learned from SARS and MERS epidemic. *Asian Pac J Allergey Immunol* **38,** 1-9, doi:10,12932/AP-200220-0772 (2020).
